# Supplementary material for: Sleep and motor learning in stroke (SMiLES): a longitudinal study investigating sleep-dependent consolidation of motor sequence learning in the context of recovery after stroke
Source: BMJ Open. 2024 Feb 13;14(2):e077442. doi: 10.1136/bmjopen-2023-077442 (PMC10868290; doi:10.1136/bmjopen-2023-077442)
Supplement: Supplementary data [file bmjopen-2023-077442supp001.pdf]

Supplementary File 1: Button box technical note

Overview

The button box was designed with a button layout based on the specifications given by Gudberg, Wulf & Johansen-Berg[1] using commercially available parts.
Following initial evaluation, the design was revised to reduce button noise to minimise any potential for interference.
The button box communicates with the experiment software via a programmable USB-Human Interface Device (HID) module. This allows the buttons to be presented as any keyboard or joystick button required by the experiment software without the need for any additional software or drivers and is compatible with all modern operating systems.

Construction

The button box is built around an appropriately sized aluminium instrument case (CamdenBoss RTM2703-BR/B, RS stock no. 170-7825) with a sloped front on which four 60mm buttons (low-profile domed arcade buttons, purchased from Arcade World UK) are mounted according to the layout described in Gudberg et al.[1] such that they can be easily operated by the participant while resting their elbow on a flat surface, if needed (Figure 1).
The buttons are connected to a programmable USB HID interface board (Ultimarc U-HID Nano) which is wrapped with heatshrink to provide insulation and mechanical protection and mounted internally to the box. The module's USB interface is then connected to a panel mount USB B socket (RS stock no. 874-1248) on the rear of the case via a short USB cable secured in place with heatshrink.

Noise reduction

The following steps were taken to minimise acoustic noise from the buttons:
The case was lined with sound deadening material (Teroson Adhesive bitumen acoustic insulation, RS stock no. 369-4715) in order to reduce the transmission of acoustic noise via the case.
The stock microswitches provided with the buttons were replaced with a quieter type (Cherry D44X, purchased from Arcade World UK) to reduce noise caused by switch actuation.
Thin self-adhesive foam was added to the back of the button plungers to reduce noise caused by impact of the plunger against the button housing.

A layer of the same sound deadening material used to line the case was applied to the back of the light diffuser and the opposing surface inside the button plunger to deaden sound conducted through the plunger.

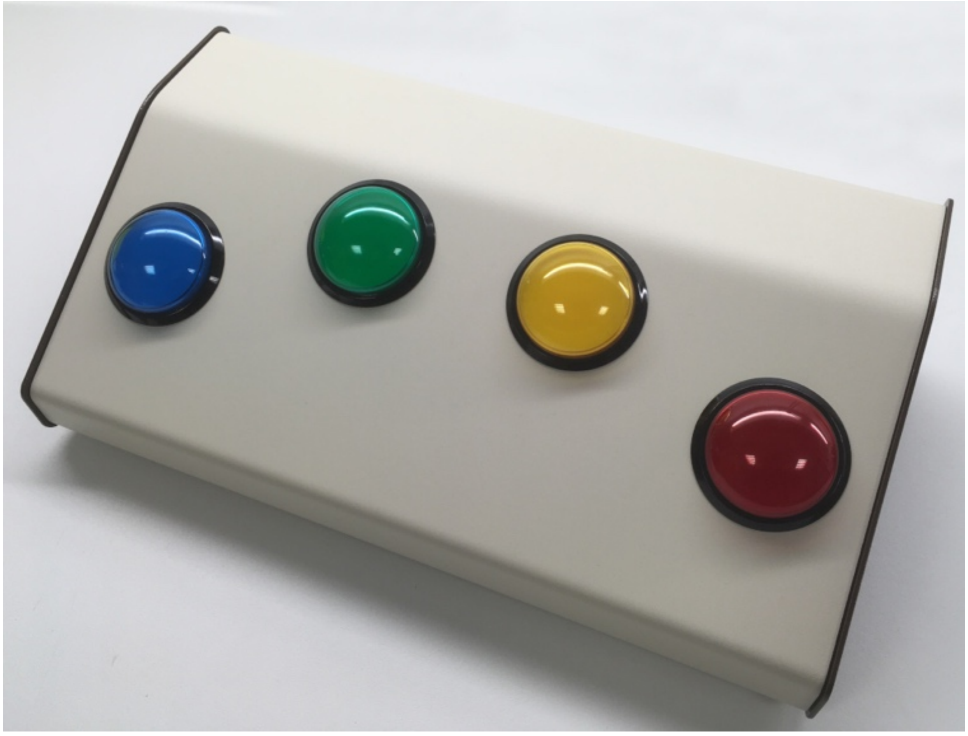

Figure 1: Image of the button box

References

Gudberg, C., Wulff, K., & Johansen-Berg, H. (2015). Sleep-dependent motor memory consolidation in older adults depends on task demands. Neurobiology of aging, 36(3), 1409–1416. doi: https://doi.org/10.1016/j.neurobiolaging.2014.12.014
