# Supplementary material for: Sleep and motor learning in stroke (SMiLES): a longitudinal study investigating sleep-dependent consolidation of motor sequence learning in the context of recovery after stroke
Source: BMJ Open. 2024 Feb 13;14(2):e077442. doi: 10.1136/bmjopen-2023-077442 (PMC10868290; doi:10.1136/bmjopen-2023-077442)
Supplement: Author's manuscript [file bmjopen-2023-077442.draft_revisions.pdf]

BMJ Open

BMJ Open is committed to open peer review. As part of this commitment we make the peer review history of every article we publish publicly available.

When an article is published we post the peer reviewers' comments and the authors' responses online. We also post the versions of the paper that were used during peer review. These are the versions that the peer review comments apply to.

The versions of the paper that follow are the versions that were submitted during the peer review process. They are not the versions of record or the final published versions. They should not be cited or distributed as the published version of this manuscript.

BMJ Open is an open access journal and the full, final, typeset and author-corrected version of record of the manuscript is available on our site with no access controls, subscription charges or pay-per-view fees (http://bmjopen.bmj.com).

If you have any questions on BMJ Open's open peer review process please email info.bmjopen@bmj.com

BMJ Open

Sleep and motor learning in stroke (SMiLES) - a longitudinal study investigating sleep-dependent consolidation of motor sequence learning in the context of recovery after stroke

Journal:	*BMJ Open*
Manuscript ID	bmjopen-2023-077442
Article Type:	Protocol
Date Submitted by the Author:	05-Jul-2023

Complete List of Authors:	Weightman, Matthew; University of Oxford, Wellcome Centre for Integrative Neuroimaging (WIN), Nuffield Department of Clinical Neurosciences<br>Robinson, Barbara; University of Oxford, Wellcome Centre for Integrative Neuroimaging (WIN), Nuffield Department of Clinical Neurosciences<br>Mitchell, Morgan ; University of Oxford, Wellcome Centre for Integrative Neuroimaging (WIN), Nuffield Department of Clinical Neurosciences<br>Garratt, Emma; Oxford Health NHS Foundation Trust, Oxfordshire Stroke Rehabilitation Unit (OSRU)<br>Teal, Rachel; Oxford University Hospitals NHS Foundation Trust, MRC Stroke Unit, Oxford Centre for Enablement<br>Rudgewick-Brown, Andrew; University of Oxford, Wellcome Centre for Integrative Neuroimaging (WIN), Nuffield Department of Clinical Neurosciences<br>Demeyere, Nele; Oxford University, Wolfson Centre for the Prevention of Stroke and Dementia, Nuffield Department of Clinical Neurosciences<br>Fleming, Melanie K. ; University of Oxford, Wellcome Centre for Integrative Neuroimaging (WIN), Nuffield Department of Clinical Neurosciences<br>Johansen-Berg, Heidi; University of Oxford, Wellcome Centre for Integrative Neuroimaging (WIN), Nuffield Department of Clinical Neurosciences

Keywords:	Stroke < NEUROLOGY, REHABILITATION MEDICINE, NEUROPHYSIOLOGY

SCHOLARONE™
Manuscripts

Sleep and motor learning in stroke (SMiLES) - a longitudinal study investigating sleep-dependent consolidation of motor sequence learning in the context of recovery after stroke

Authors: Matthew Weightman[1], Barbara Robinson[1], Morgan P Mitchell[1], Emma Garratt[2], Rachel Teal[3], Andrew Rudgewick-Brown[1], Nele Demeyere[4], Melanie K Fleming[1^], Heidi Johansen-Berg[1^]

^ These authors contributed equally

Affiliations:

[1]Wellcome Centre for Integrative Neuroimaging (WIN), Nuffield Department of Clinical Neurosciences, University of Oxford, Oxford, OX3 9DU, UK

[2]Oxfordshire Stroke Rehabilitation Unit (OSRU), Oxford Health NHS Foundation Trust, Oxford, United Kingdom, OX14 1AG

[3]MRC Stroke Unit, Oxford Centre for Enablement, Oxford University Hospitals NHS Foundation Trust, Oxford, United Kingdom, OX3 7HE

[4]Wolfson Centre for the Prevention of Stroke and Dementia, Nuffield Department of Clinical Neurosciences, University of Oxford, Oxford, OX3 9DU

For peer review only

Corresponding Author:
Matthew Weightman
Wellcome Centre for Integrative Neuroimaging (WIN),
Nuffield Department of Clinical Neurosciences,
University of Oxford,
Oxford,
OX3 9DU

matthew.weightman@ndcn.ox.ac.uk

Word Count: 3820

Keywords: *Stroke, Rehabilitation, Motor Consolidation, Sleep*

For peer review only

Abstract

Introduction: There is growing evidence that sleep is disrupted after stroke, with worse sleep relating to poorer motor outcomes. It is also widely acknowledged that consolidation of motor learning, a critical component of post-stroke recovery, is sleep-dependent. However, whether the relationship between disrupted sleep and poor outcomes after stroke is related to direct interference of sleep-dependent motor consolidation processes, is currently unknown. Therefore, the aim of the present study is to understand whether measures of motor consolidation mediate the relationship between sleep and clinical motor outcomes post-stroke.

Methods and Analysis: We will conduct a longitudinal observational study of up to 150 participants diagnosed with stroke affecting the upper limb. Participants will be recruited and assessed within 7 days of their stroke and followed up at approximately 1 and 6 months. The primary objective of the study is to determine whether sleep in the sub-acute phase of recovery explains the variability in upper limb motor outcomes after stroke (over and above predicted recovery potential from the PREP2 algorithm) and whether this relationship is dependent on consolidation of motor learning. We will also test whether motor consolidation mediates the relationship between sleep and whole-body clinical motor outcomes, whether motor consolidation is associated with specific electrophysiological sleep signals, and sleep alterations during sub-acute recovery.

Ethics and Dissemination: This trial has received both Health Research Authority (HRA), Health and Care Research Wales (HCRW), and National Research Ethics Service approval (IRAS: 304135; REC: 22/LO/0353). The results of this trial will help to enhance our understanding of the role of sleep in recovery of motor function after stroke and will be disseminated via presentations at scientific conferences, peer-

For peer review only

reviewed publication, public engagement events, stakeholder organisations, and other forms of media where appropriate.

Trial Registration: ClinicalTrials.gov: NCT05746260, registered on 27th February 2023.

Strengths and Limitations

- Longitudinal study, with early assessment (< 7 days post-stroke) and follow-ups throughout sub-acute recovery.
- All assessments carried out where the patient is currently residing to reduce burden.
- Inclusion of patients without capacity to consent to increase the diversity of study sample.
- Some patients may not be able to complete all aspects of the study due to level of impairment.

For peer review only

Introduction

Stroke is a leading cause of worldwide disability, with over 50% of stroke survivors experiencing long-term motor impairment - especially of the upper limb.[1-3] It is widely believed that good recovery of movement after stroke is dependent on motor learning.[4] Indeed, most current rehabilitative practises aiming to improve motor function after stroke place a key focus on learning new, or relearning old, ways in which to successfully carry out activities of daily living.[5,6] Motor learning is thought to be especially important for post-stroke recovery due to its capacity to drive structural and functional changes in the brain, termed neuroplasticity.[7,8] As good rehabilitation outcomes after stroke are dependent on the ability of the brain to learn and reorganise, it is critical we understand how neuroplasticity occurs and how it can be influenced in the context of recovery.

A key element of our understanding of neuroplasticity – which has yet to be fully explored in the context of stroke rehabilitation - is consolidation of memories during sleep.[4] Improvements in motor learning are known to not only occur online (during practise itself) but also offline, during periods of rest.[9] For some time now, sleep has been proposed to be actively engaged in the offline consolidation of motor memories.[10-12] The reactivation of task-related neural activity during sleep is believed to underpin offline memory processing, by transforming labile memory traces into persistent representations, readily available for retrieval.[13,14] These reactivations are also closely linked to fine-tuned interactions between electrophysiological signatures of slow wave sleep, namely; neocortical slow oscillations and thalamocortical sleep spindles, known to be key correlates of memory consolidation.[15-18] Taken together, this evidence highlights the importance of sleep to the offline consolidation of motor memories.

For peer review only

For peer review only - http://bmjopen.bmj.com/site/about/guidelines.xhtml

Notwithstanding, sleep disruption is commonplace after stroke,[19-22] and evidence suggests that poor sleep after brain injury, including stroke, is often associated with worse motor/cognitive impairments, quality of life, and mood.[22-24] Furthermore, we and others have demonstrated that sleep disruption during rehabilitation after stroke and/or brain injury is associated with slower functional recovery and worse motor outcomes.[23,25] However, whether this relationship exists because impaired sleep post-stroke directly disrupts memory consolidation processes, or reflects other factors that influence sleep and clinical outcomes e.g., depression, motivation etc. is yet unknown. Given that clinical gains during therapy are likely to depend on both improvements during training and sleep, it is reasonable to suggest that poor motor outcomes may be, at least in part, due to disrupted sleep and thereby associated memory processes.

Trial Objectives

Considering the link between sleep disruption and recovery, the aim of the present study is to understand to what extent behavioural measures of motor consolidation mediate the relationship between sleep quality and clinical motor outcomes post-stroke. We will investigate whether sleep measures in the sub-acute phase explain variation in motor outcomes (over and above variation explained by baseline assessment, and other covariates) and whether this relationship is mediated by variation in behavioural measures of overnight consolidation. More specifically, we aim to:

Primary Objectives

For peer review only - http://bmjopen.bmj.com/site/about/guidelines.xhtml

1) Test whether sleep measures explain variability in recovery of movement of the upper limb.
2) Assess whether the relationship between sleep and recovery depends on motor consolidation.

Secondary Objective

1) Determine if motor consolidation at 1-month post-stroke mediates the relationship between sleep and clinical outcomes, such as whole-body motor impairment, mobility, and hand function.

Exploratory Objectives

1) Explore whether motor consolidation is associated with specific electrophysiological markers of sleep after stroke.
2) Investigate changes in sleep alongside recovery.
3) Explore correlations between sleep disruption and motor function.

Methods and Analysis

Participant Recruitment

Up to 150 participants, diagnosed with stroke affecting the upper limb, will be recruited. All participants will be identified from stroke wards at the acute stage post stroke (≤ 7 days). Patients will be approached by a member of the clinical team or a clinical research facilitator (e.g., research nurse) to determine potential interest and provide information about the study. Upon verbal consent (or in case of expressive speech problems, a clear non-verbal indication e.g., a nod), a researcher will approach interested patients to go through the informed consent process for the study. It will be made clear that participation is voluntary and will not affect their medical or rehabilitative care. Where the mental capacity to provide informed

For peer review only

For peer review only - http://bmjopen.bmj.com/site/about/guidelines.xhtml

consent is in doubt, a mental capacity assessment for consenting to the research study will be conducted and appropriate consultee advice will be sought from a familial carer, other relative, or close friend if appropriate (Figure 1). Inclusion and exclusion criteria are shown in Table 1. The trial was registered on February 27th, 2023 (prior to recruitment of the first participant; NCT05746260), opened to recruitment in March 2023, with a planned trial end date of March 2027.

[Insert Figure 1]

Figure 1: *Consent process, including capacity to consent decision framework.*

<u>Sample Size Calculations</u>

Based on findings from previous work in inpatients with brain injury,[23] we predict that sleep measures increase variance in motor outcomes explained by ~10% and therefore estimate a sample size of n = 100 will be required. In anticipation of participant attrition, we will aim to recruit 150 participants.

[Insert Table 1]

Table 1: *Inclusion and exclusion criteria*

Trial Design

The trial will be conducted as a longitudinal observational study. The direct clinical care team or research nurse will identify and screen potential participants, checking their medical history to confirm eligibility. Should participants be interested and meet all eligibility criteria, a researcher will then arrange a consultation for the baseline

For peer review only

visit (≤ 7 days post-stroke), where they will go over the details of the study, answer any remaining questions and complete the informed consent process (Figure 1). Baseline assessment will be conducted as soon as possible thereafter. Following consent, participants will be enrolled in the trial and assigned a unique study code, and the direct care team will be informed (verbally) that the patient has consented to take part in the study. We will also inform each participant's GP of consent in the study.

[Insert Figure 2]

Figure 2: *Flowchart of the trial timeline and assessments. BMI, Body Mass Index; SAFE, Shoulder Abduction Finger Extension; NIHSS, National Institute of Health Stroke Scale; SCI, Sleep Condition Indicator; PHQ-8, Patient Health Questionnaire; STOP-Bang, Obstructive Sleep Apnoea Questionnaire; SRTT, Serial Reaction Time Task; EEG, Electroencephalography; ARAT, Action Research Arm Test; FMA, Fugl-Meyer Assessment; RMI, Rivermead Mobility Index; BBT, Box and Blocks Test; mRS, modified Rankin Scale.*

Outcome Measures

Our main outcomes will be used to determine whether sleep in the sub-acute phase of recovery after stroke explains the variability seen in upper limb motor outcomes and whether this relationship is dependent on overnight consolidation of motor memories. The main outcome measures are described in detail in the descriptions of assessment sessions below. Briefly:

Sleep disruption will be measured via 7-night actigraphy recordings and sleep diaries, indicating estimated total sleep time, wakefulness after sleep onset (WASO), and sleep fragmentation.

For peer review only

Symptoms of insomnia will be recorded using the Sleep Condition Indicator questionnaire (SCI),[26,27] with higher scores indicating fewer symptoms of insomnia (range 0-32).

Motor consolidation will be measured at approximately 1-month after stroke and will describe the change in performance (response time/accuracy) from evening training to morning retest in the adapted Serial Reaction Time Task (SRTT)[12,28].

Upper limb function will be assessed at approximately 6 months post-stroke using the Action Research Arm Test.[29] Scores range between 0-57, with higher scores indicates better upper limb ability.

Secondary outcome measures include: whole body motor impairment, hand dexterity, and mobility at ~ 6 months after stroke, assessed via the Fugl-Meyer Assessment (FMA),[30] Nine Hole Peg Test (9HPT)[31] and Rivermead Mobility Index (RMI),[32] respectively (higher scores across all measurements indicates better movement/performance). These outcomes will help to determine whether motor consolidation mediates the relationship between sleep and different aspects of motor recovery. Actigraphy derived sleep measures and SCI scores at 1- and 6-months post-stroke will be used to explore changes in sleep quality during sub-acute recovery. Additionally, electroencephalogram (EEG) derived sleep measures (e.g., slow oscillation amplitude, sleep spindle density, slow oscillation-sleep spindle coupling etc) collected in a sub-set of participants at 1-month post-stroke will be used to discern whether consolidation of motor learning is associated with specific sleep signals in this population.

Other pre-specified measures include: symptoms of depression via the patient health questionnaire (PHQ-8),[33] stroke severity/disability via the modified Ranking Scale (mRS), and physical activity (actigraphy recordings) assessed at

For peer review only

For peer review only - http://bmjopen.bmj.com/site/about/guidelines.xhtml

approximately 1 and 6 months post-stroke, in addition to the risk of obstructive sleep apnoea (OSA) using the STOP-Bang questionnaire[34] and presence of sleep disorders[35] at 1-month post-stroke. These will be used as covariates in analyses as appropriate.

Baseline Assessment

The baseline assessment will take place ≤ 7 days post-stroke. We will record participant demographics, including: age, sex, ethnicity, weight, height, stroke type, stroke date, and current medications (Figure 2).

To characterise expected upper limb recovery potential, we will follow procedures for the Predict Recovery Potential (PREP2) algorithm.[36] In the initial days post-stroke, the PREP2 algorithm combines neurological biomarkers and clinical measures, to predict upper limb functional outcomes at 3 months (Figure 3). Patients can be categorised into one of four different recovery categories: excellent, good, poor, and limited, with predictions shown to be accurate for up to 80% of patients.[36,37] A shoulder abduction and finger extension (SAFE) score will be calculated using the Medical Research Council (MRC) grades to determine participants' strength in each of these movements. Movements are scored between 0 and 5, where 0 represents no palpable muscle activity and 5 describes normal strength and range of movement. Participants with a SAFE score of < 5 will require transcranial magnetic stimulation (TMS) to establish the presence of motor evoked potentials (MEPs) in the paretic upper limb for further categorisation. Participants will be screened separately for TMS safety and participants can still take part in the study if unable to undergo TMS. If a participant requires TMS - and is deemed appropriate to do so as per the screening checklist - we will use surface

For peer review only

electromyography (EMG) to record muscle activity from the extensor carpi radialis (ECR) and first dorsal interosseus (FDI) of the affected upper limb between 3-7 days after stroke. A patient will be classified as MEP+ if MEPs of any amplitude are elicited with a consistent latency in response to at least 5 stimuli at rest or during voluntary muscle facilitation and MEP- if no MEPs can be elicited at 100% maximum stimulator output (MSO) either at rest or while attempting voluntary muscle facilitation. We estimate that approximately 30% of participants will require TMS.[36] If a participant is unable to undergo TMS, we will use all other biomarker information available and categorise on a 'worst case scenario' basis (analyses will be conducted with and without these participants included to ensure that this estimation does not influence findings). Age and National Institute for Health Stroke Scale (NIHSS) scores will also be collected to dictate final categorisation.

[Insert Figure 3]

Figure 3: *PREP2 algorithm, included with permission, under license CC BY-NC-ND 4.0 (https://presto.auckland.ac.nz).[36] The algorithm uses neurological and clinical biomarkers to predict four different categories of potential recovery outcomes at 3 months after stroke. SAFE, Shoulder Abduction Finger Extension score; MEP, Motor Evoked Potential; NIHSS, National Institute of Health Stroke Scale.*

<u>1-month post-stroke</u>

All procedures involved in the 1-month visit will be conducted at the location the patient is currently residing, with researchers visiting to collect data and set up equipment. At ~1-month after stroke, sleep measures will be collected via 7-night actigraphy recordings (measuring day/night activity, estimated total sleep time, WASO, and sleep fragmentation index) and completion of the SCI).[26] For actigraphy

For peer review only - http://bmjopen.bmj.com/site/about/guidelines.xhtml

measures, participants will wear a waterproof monitor on their least affected wrist (MotionWatch 8, CamNTech). A sleep diary, detailing approximately what time participants fell asleep and woke up for each of the 7 nights, will also be completed (Figure 2). We have previously, and continue to, successfully implement similar actigraphy and diary-based approaches in multiple research projects comprising of over 200 patients, proving to be safe and tolerable.[22,23,38,39] We will assess the presence of other sleep disorders via completion of a sleep screening and STOP-Bang questionnaires, and mood by means of the PHQ-8. Additionally, we will ask participants to complete a series of questions to determine the score for the mRS, and record current medications. We will record where the participant is residing at the time of the visit (e.g., home, rehabilitation unit, care home etc.), and ask participants to self-report whether they have received motor rehabilitation and/or sleep therapy following their stroke, and if so, approximately how much (e.g., daily, weekly).

We will assess consolidation of motor sequence learning using an adapted version of the SRTT, see Figure 2. The task has been adapted so that participants are required to move their entire upper limb and press each button with their whole hand rather than individual fingers as is customary in SRTTs,[12] see supplementary file 1. Studies using similar tasks have reported effective learning in stroke patients using the paretic upper limb[40] and sleep-dependent motor improvements in older adult participants.[12] Participants will learn to press buttons on a button box with their affected limb following a repeated 8-item sequence. On-screen representations of each button will become highlighted, indicating which button to press. Once a response is given the next target cue in the sequence will become highlighted, and so on, regardless of whether the correct response is given. Participants will perform

For peer review only - http://bmjopen.bmj.com/site/about/guidelines.xhtml

two different versions of the task (training and test), one in the evening and then one the following morning, respectively (Figure 2). The training task will include 20 blocks of trials, with three repetitions of the 8-item sequence in each block. The test, aimed to determine sleep-dependent changes, will only include 3 blocks of trials to avoid confounds related to additional learning. To probe sequence specific learning, two blocks of 'random' sequences will be inserted at the beginning and towards the end of each the training and test tasks. We will not explicitly inform participants of the sequence structure, however, a banner will appear at the top of the screen during every block, indicating whether the button presses will follow the sequence or a random order. At the end of the test task, participants will be asked to attempt to replicate as much of the sequence order as they can remember/were aware of without being shown the cues. Participants will be asked to train on the task between 8-10 pm or approximately 30-60 minutes before they intend to go to sleep and then be tested the following morning between 8-10am or at least 30 minutes after wake. The training will take approximately 20-30 minutes and retest will take no more than 10 minutes. Measures of both speed (response times) and accuracy will be recorded.

In a subset of participants, we will also measure overnight brain activity using the commercial, portable low-density EEG device; Dreem headband (DreemTM, France). Evidence suggests that Dreem headbands are efficacious for monitoring sleep-related electrophysiological signals and are comparable to gold-standard polysomnography (PSG) protocols.[41] We have also previously explored the feasibility of use by stroke survivors at home (unpublished data), with no major issues reported. Participants will be asked to wear the Dreem headband for three consecutive nights: one acclimatisation night followed by the night prior to

For peer review only - http://bmjopen.bmj.com/site/about/guidelines.xhtml

completing the motor learning task, and the night of the motor learning task. Only participants who are residing at home will be invited for this assessment and they may opt out with no consequence to any of the other aspects of the study.

<u>6 months post-stroke</u>

We will assess sleep using the same protocol as detailed for the 1-month visit (SCI, 7-night actigraphy, and daily sleep diary). Similarly, we will measure mood (PHQ-8), disability (mRS), and record current medications (Figure 2). We will also document where the participant is residing, and self-report of whether they have received motor rehabilitation and/or sleep therapy (and if so, how much) since the previous assessment.

To determine functional recovery 6 months after stroke, we will carry out a number of standardised clinical assessments requiring participants to make everyday movements with their hands, arms, and legs. These include the ARAT, upper and lower extremity sections of the FMA, the RMI and the 9HPT. Again, researchers will visit participants in their homes or where they are currently residing to carry out all aspects of the 6-month assessment. In instances where participants prefer not to complete the movement assessments at their residing location, they will be invited to attend the research lab to complete these.

Data Management

Direct access to data will be granted to authorised representatives from the trial sponsor and host institution for monitoring and/or audit of the study in compliance with regulations. Participants will be assigned a unique study code, which will be used on all study documents (apart from consent forms) and all documents will be

For peer review only

stored safely in confidential conditions. Anonymous data will be shared openly through a data sharing initiative (e.g., Open Science Framework; OSF) following publication or public release of the study results.

Data/Statistical Analyses

Primary Analyses

To test whether sleep in the sub-acute phase of recovery after stroke explains variability seen in upper limb motor outcomes, we will use the general linear model to test the association between sleep measures at 1 month and ARAT score at 6-months post-stroke. We will include covariates such as anticipated recovery outcome category (from PREP2 algorithm), PHQ-8 score and the activity metric from actigraphy recordings as appropriate. To determine whether this relationship depends on motor consolidation, we will test for a mediation effect of motor consolidation at 1 month on this association.

Secondary Analyses

To test whether motor consolidation mediates the relationship between sleep and a broader range of clinical outcomes and timepoints, we will use the general linear model, testing for a mediation effect of motor consolidation at 1-month post-stroke on the correlation between sleep measures at 1-month and ARAT, FMA, 9HPT, and RMI scores at 6 months post-stroke.

Exploratory Analyses

For peer review only

For peer review only - http://bmjopen.bmj.com/site/about/guidelines.xhtml

To explore whether motor consolidation is associated with specific sleep signals after stroke, we will use the general linear model to correlate motor consolidation with EEG derived sleep metrics at 1-month post-stroke. Finally, to explore changes in sleep alongside recovery we will use the general linear model to test for changes in sleep measures from 1-month to 6 months post-stroke.

Study Monitoring

The study management group, consisting of the principal investigator and members of the research team, will meet multiple times per year to monitor the progress of the study. Participants will be regularly monitored, and data evaluated to check for protocol compliance and accuracy in relation to source documents. Responsible personnel from the NHS Trust(s) and/or sponsor may also monitor or audit the trial where appropriate.

Throughout the trial timeline, we will check with participants for the occurrence of any adverse events (AEs). Potential expected minor AEs include distress associated with completing mood questionnaires and skin irritation from wearing the actigraphy monitor. Any AEs that do occur during study enrolment will be documented and participants will be advised on the most appropriate course of action, e.g., withdrawing from particular assessments or contacting their General Practitioner (GP). There is a potential, but very small, risk of seizure with TMS. The most recent update to safety guidelines suggested that, given the large number of patients who have received TMS and the small number of seizures, the risk of TMS to induce seizures is very low.[42] All participants who require TMS will be screened prior to testing and all procedures will be conducted within the safety guidelines. There are no anticipated serious AEs (SAEs) associated with the present trial. Any SAEs that

For peer review only

occur (and deemed unexpected and related to research procedures) will be reported to the appropriate ethics committee within 15 days of awareness of the event, using the Health Research Authority (HRA) report of SAE form (see HRA website for further info).

During the course of the trial a participant may choose to withdraw at any time point and for any reason. The reason for withdrawal will be recorded if known, but participants are not obliged to provide a reason. Data from withdrawn participants may be kept and used for analyses as appropriate. If there is sufficient time to conduct all necessary study assessments within the study period, additional participants will be recruited to replace withdrawals. If a participant is unable to comply with one aspect of the assessment (e.g., the motor learning task), then they may continue other assessments unless they choose to withdraw entirely. Moreover, an investigator may choose to discontinue a participant from the trial if it is considered necessary for any reason e.g., significant protocol deviation, non-compliance, ineligibility (either arising during the study period or retrospectively having been overlooked at screening) etc.

Patient and Public Involvement

Stroke survivors were involved in producing and reviewing all participant facing documents. We also encouraged patients to give feedback on the motor learning task at different public/patient engagement events throughout the design of the study and during pilot testing. This feedback was used to write instructions and edit task difficulty. We will seek further assistance from patient/public contributors when disseminating results from the trial - in the form of lay summaries - to ensure key messages are communicated in a meaningful way to stroke communities.

For peer review only

Ethics and Dissemination

This trial has received both Health Research Authority (HRA), Health and Care Research Wales (HCRW), and National Research Ethics Service approval (IRAS: 304135; REC: 22/LO/0353). The study is registered on a publicly accessible database (clinicaltrials.gov: NCT05746260). Results of the study will be uploaded to clinicaltrials.gov within 12 months of the end of trial and disseminated via presentations at scientific conferences, peer-reviewed publication, public engagement events, stakeholder organisations, and other forms of media where appropriate.

Summary and Conclusions

The present trial aims to investigate the relationship between sleep, motor consolidation, and clinical outcomes after stroke. The importance of motor learning and neuroplasticity in post-stroke recovery is widely recognised,[4,8,43] however, the role of sleep in offline consolidation of motor memories is an area that has yet to be fully explored in the context of stroke rehabilitation. We will investigate whether sleep measures in the sub-acute stage of stroke explain variability in recovery of upper limb function, assessing the relationship between sleep and recovery in terms of motor consolidation, and determining whether motor consolidation mediates the relationship between sleep and clinical outcomes. Using wearable remote technologies, we will also explore electrophysiological markers of sleep after stroke, changes in sleep alongside recovery, and correlations between sleep and motor function. We aim to build on our previous work showing that sleep is disrupted after stroke/brain injury and predicts poorer recovery,[22,23] to determine whether the

For peer review only

consolidation of motor memories is a candidate process to mediate this relationship. We hope the findings of this study will help to enhance our understanding of the role of sleep in stroke recovery and may inform the development of novel interventions for improving post-stroke rehabilitation outcomes.

For peer review only

Acknowledgements

We would like to thank the individuals at OSRU and NIHR CRN Stroke for their support in setting up, recruiting for, and facilitating the trial. Thanks also to Sebastian Rieger for his input regarding behavioural motor task hardware design.

Author Contributions

MW, BR, MKF, and HJB conceived and designed the protocol with input from MM. EG, RT and ND facilitate recruitment, consent process, and data collection. ARB designed and constructed the hardware for the behavioural motor task. MW and BR are involved in the day-to-day running of the trial. MW drafted the manuscript. All authors edited, revised and approved the final version of the manuscript.

Funding

This study is funded by the Wellcome Trust (Principal Research Fellowship to HJB; 222446/Z/21/Z) and supported by the NIHR Oxford (Oxford University Hospitals) and Oxford Health Biomedical Research Centres (BRC). These funding sources had no role in the design of this study and will not have any role during its execution, analyses, or interpretation of data. The Wellcome Centre for Integrative Neuroimaging is supported by core funding from the Wellcome Trust.

Conflicting Interests

No conflicting interests declared.

Rights Retention

For peer review only

This research is funded in whole, or in part, by the Wellcome Trust [Grant number 222446/Z/21/Z]. For the purpose of open access, the author has applied a CCBY public copyright license to any Author Accepted Manuscript version arising from this submission.

For peer review only

References

1) Wolfe, C. D. (2000). The impact of stroke. *British medical bulletin*, *56*(2), 275–286. doi: https://doi.org/10.1258/0007142001903120

2) Raghavan, P. (2015). Upper limb motor impairment after stroke. *Physical Medicine and Rehabilitation Clinics*, *26*(4), 599–610. doi: https://doi.org/10.1016/j.pmr.2015.06.008

3) Feigin, V. L., Brainin, M., Norrving, B., Martins, S., Sacco, R. L., Hacke, W., . . . Lindsay, P. (2022). World stroke organization (WSO): global stroke fact sheet 2022. *International Journal of Stroke*, *17*(1), 18–29. doi: https://doi.org/10.1177/17474930211065917

4) Krakauer, J. W. (2006). Motor learning: its relevance to stroke recovery and neurorehabilitation. Current opinion in neurology, 19(1), 84–90. doi: 10.1097/01.wco.0000200544.29915.cc

5) Winstein, C., Lewthwaite, R., Blanton, S. R., Wolf, L. B., & Wishart, L. (2014). Infusing motor learning research into neurorehabilitation practice: a historical perspective with case exemplar from the accelerated skill acquisition program. *Journal of neurologic physical therapy: JNPT*, *38*(3), 190. doi: 10.1097/NPT.0000000000000046

6) Leech, K. A., Roemmich, R. T., Gordon, J., Reisman, D. S., & Cherry-Allen, K. M. (2021). Updates in motor learning: Implications for physical therapist practice and education. Physical therapy. doi: https://doi.org/10.1093/ptj/pzab250

7) Dayan, E., & Cohen, L. G. (2011). Neuroplasticity subserving motor skill learning. *Neuron*, *72*(3), 443–454. doi: https://doi.org/10.1016/j.neuron.2011.10.008

8) Sampaio-Baptista, C., Sanders, Z.-B., & Johansen-Berg, H. (2018). Structural plasticity in adulthood with motor learning and stroke rehabilitation. *Annual review of neuroscience, 41, 25–40. doi: https://doi.org/10.1146/annurev-neuro-080317-062015*

9) Robertson, E. M., Pascual-Leone, A., & Miall, R. C. (2004). Current concepts in procedural consolidation. *Nature Reviews Neuroscience*, *5*(7), 576–582. doi: https://doi.org/10.1038/nrn1426

10) Walker, M. P., Brakefield, T., Seidman, J., Morgan, A., Hobson, J. A., & Stickgold, R. (2003). Sleep and the time course of motor skill learning. *Learning & memory*, *10*(4), 275–284. doi: 10.1101/lm.58503

11) Stickgold, R., & Walker, M. P. (2007). Sleep-dependent memory consolidation and reconsolidation. *Sleep medicine*, *8*(4), 331–343. doi: https://doi.org/10.1016/j.sleep.2007.03.011

For peer review only

For peer review only - http://bmjopen.bmj.com/site/about/guidelines.xhtml

12) Gudberg, C., Wulff, K., & Johansen-Berg, H. (2015). Sleep-dependent motor memory consolidation in older adults depends on task demands. Neurobiology of aging, 36(3), 1409–1416. doi: https://doi.org/10.1016/j.neurobiolaging.2014.12.014

13) Rasch, B., & Born, J. (2013). About sleep's role in memory. *Physiological reviews*. doi: https://doi.org/10.1152/physrev.00032.2012

14) Klinzing, J. G., Niethard, N., & Born, J. (2019). Mechanisms of systems memory consolidation during sleep. Nature neuroscience, 22(10), 1598–1610. doi: https://doi.org/10.1038/s41593-019-0467-3

15) Morin, A., Doyon, J., Dostie, V., Barakat, M., Tahar, A. H., Korman, M., ... Carrier, J. (2008). Motor sequence learning increases sleep spindles and fast frequencies in post-training sleep. *Sleep*, *31*(8), 1149–1156. doi: https://doi.org/10.5665/sleep/31.8.1149

16) Born, J., & Wilhelm, I. (2012). System consolidation of memory during sleep. *Psychological research*, *76*, 192–203. doi: https://doi.org/10.1007/s00426-011-0335-6

17) Ramanathan, D. S., Gulati, T., & Ganguly, K. (2015). Sleep-dependent reactivation of ensembles in motor cortex promotes skill consolidation. *PLoS biology*, *13*(9), e1002263. doi: https://doi.org/10.1371/journal.pbio.1002263

18) Fogel, S., Albouy, G., King, B. R., Lungu, O., Vien, C., Bore, A., . . . Doyon, J. (2017). Reactivation or transformation? Motor memory consolidation associated with cerebral activation time-locked to sleep spindles. *PloS one*, *12*(4), e0174755. doi: https://doi.org/10.1371/journal.pone.0174755

19) Duss, S. B., Seiler, A., Schmidt, M. H., Pace, M., Adamantidis, A., Müri, R. M., & Bassetti, C. L. (2017). The role of sleep in recovery following ischemic stroke: a review of human and animal data. *Neurobiology of sleep and circadian rhythms*, *2*, 94–105. doi: https://doi.org/10.1016/j.nbscr.2016.11.003

20) Sterr, A., Kuhn, M., Nissen, C., Ettine, D., Funk, S., Feige, B., . . . Riemann, D. (2018). Post-stroke insomnia in community-dwelling patients with chronic motor stroke: Physiological evidence and implications for stroke care. *Scientific Reports*, *8*(1), 1–9. doi: https://doi.org/10.1038/s41598-018-26630-y

21) Khot, S. P., & Morgenstern, L. B. (2019). Sleep and stroke. Stroke, 50(6), 1612–1617. doi: https://doi.org/10.1161/STROKEAHA.118.023553

22) Fleming, M. K., Smejka, T., Henderson Slater, D., Chiu, E. G., Demeyere, N., & Johansen- Berg, H. (2021). Self-reported and objective sleep measures in stroke survivors with incomplete motor recovery at the chronic stage. *Neurorehabilitation and Neural Repair*, *35*(10), 851–860. doi: https://doi.org/10.1177/15459683211029889

23) Fleming, M. K., Smejka, T., Henderson Slater, D., van Gils, V., Garratt, E., Yilmaz Kara, E., & Johansen-Berg, H. (2020). Sleep disruption after brain injury is

For peer review only

For peer review only - http://bmjopen.bmj.com/site/about/guidelines.xhtml

associated with worse motor outcomes and slower functional recovery. *Neurorehabilitation and Neural Repair*, *34*(7), 661–671. doi: https://doi.org/10.1177/154596832092966

24) Fulk, G. D., Boyne, P., Hauger, M., Ghosh, R., Romano, S., Thomas, J., . . . Klingman, K. (2020). The impact of sleep disorders on functional recovery and participation following stroke: A systematic review and meta- analysis. Neurorehabilitation and Neural Repair, 34(11), 1050–1061. doi: https://doi.org/10.1177/1545968320962501

25) Iddagoda, M. T., Inderjeeth, C. A., Chan, K., & Raymond, W. D. (2020). Post-stroke sleep disturbances and rehabilitation outcomes: a prospective cohort study. Internal Medicine Journal, 50(2), 208–213. doi: https://doi.org/10.1111/imj.14372

26) Espie, C. A., Kyle, S. D., Hames, P., Gardani, M., Fleming, L., & Cape, J. (2014). The sleep condition indicator: a clinical screening tool to evaluate insomnia disorder. *BMJ open*, *4*(3), e004183. doi: doi:10.1136/bmjopen-2013-004183

27) Espie, C. A., Farias Machado, P., Carl, J. R., Kyle, S. D., Cape, J., Siriwardena, A. N., & Luik, A. I. (2018). The sleep condition indicator: reference values derived from a sample of 200 000 adults. *Journal of sleep research*, *27*(3), e12643. doi: https://doi.org/10.1111/jsr.12643

28) Robertson, E. M., Pascual-Leone, A., & Press, D. Z. (2004). Awareness modifies the skill-learning benefits of sleep. *Current biology*, *14*(3), 208–212. doi: https://doi.org/10.1016/j.cub.2004.01.027

29) Lyle, R. C. (1981). A performance test for assessment of upper limb function in physical rehabilitation treatment and research. International journal of rehabilitation research, 4(4), 483–492.

30) Fugl-Meyer, A. R., Jääskö, L., Leyman, I., Olsson, S., & Steglind, S. (1975). A method for evaluation of physical performance. *Scand. J. Rehabil. Med*, *7*(1), 13–31.

31) Mathiowetz, V., Weber, K., Kashman, N., & Volland, G. (1985). Adult norms for the nine hole peg test of finger dexterity. *The Occupational Therapy Journal of Research*, *5*(1), 24–38. doi: https://doi.org/10.1177/153944928500500102

32) Collen, F. M., Wade, D. T., Robb, G., & Bradshaw, C. (1991). The Rivermead mobility index: a further development of the Rivermead motor assessment. *International disability studies*, *13*(2), 50–54. doi: https://doi.org/10.3109/03790799109166684

33) Kroenke, K., Spitzer, R. L., & Williams, J. B. (2001). The PHQ-9: validity of a brief depression severity measure. Journal of general internal medicine, 16(9), 606–613. doi: https://doi.org/10.1046/j.1525-1497.2001.016009606.x

34) Chung, F., Abdullah, H. R., & Liao, P. (2016). Stop-bang questionnaire: a practical approach to screen for obstructive sleep apnea. *Chest*, *149*(3), 631–638. doi: https://doi.org/10.1378/chest.15-0903

For peer review only - http://bmjopen.bmj.com/site/about/guidelines.xhtml

35) Wilson, S. J., Nutt, D., Alford, C., Argyropoulos, S., Baldwin, D. S., Bateson, A., . . . others (2010). British association for psychopharmacology consensus statement on evidence-based treatment of insomnia, parasomnias and circadian rhythm disorders. Journal of psychopharmacology, 24(11), 1577–1601. doi: https://doi.org/10.1177/0269881110379307

36) Stinear, C. M., Byblow, W. D., Ackerley, S. J., Smith, M.-C., Borges, V. M., & Barber, P. A. (2017). Prep2: A biomarker-based algorithm for predicting upper limb function after stroke. *Annals of clinical and translational neurology*, *4*(11), 811–820. doi: https://doi.org/10.1002/acn3.488

37) Smith, M.-C., Ackerley, S. J., Barber, P. A., Byblow, W. D., & Stinear, C. M. (2019). Prep2 algorithm predictions are correct at 2 years poststroke for most patients. Neurorehabilitation and neural repair, 33(8), 635–642. doi: https://doi.org/10.1177/1545968319860481

38) Fleming, M. K., Smejka, T., Macey, E., Luengo-Fernandez, R., Henry, A. L., Robinson, B., . . . Johansen-Berg, H. (2023). Improving sleep after stroke: a randomised controlled trial of digital cognitive behavioural therapy for insomnia. *medRxiv*, 2023–02. doi: https://doi.org/10.1101/2023.02.07.23285580

39) Weightman, M., Robinson, B., Fallows, R., Henry, A. L., Kyle, S. D., Garratt, E., ... others (2023). Improving sleep and learning in rehabilitation after stroke, part 2 (inspires2): study protocol for a home-based randomised control trial of digital cognitive behavioural therapy (dcbt) for insomnia. *BMJ open*, *13*(4), e071764. doi: http://dx.doi.org/10.1136/bmjopen-2023-071764

40) Fleming, M. K., Newham, D. J., & Rothwell, J. C. (2018). Explicit motor sequence learning with the paretic arm after stroke. *Disability and Rehabilitation*, *40*(3), 323–328. doi: https://doi.org/10.1080/09638288.2016.1258091

41) Arnal, P. J., Thorey, V., Debellemaniere, E., Ballard, M. E., Bou Hernandez, A., Guillot, A., . . . others (2020). The dreem headband compared to polysomnography for electroencephalographic signal acquisition and sleep staging. *Sleep*, *43*(11), zsaa097. doi: https://doi.org/10.1093/sleep/zsaa097

42) Rossi, S., Antal, A., Bestmann, S., Bikson, M., Brewer, C., Brockmöller, J., ... others (2021). Safety and recommendations for TMS use in healthy subjects and patient populations, with updates on training, ethical and regulatory issues: Expert guidelines. *Clinical Neurophysiology*, *132*(1), 269–306. doi: https://doi.org/10.1016/j.clinph.2020.10.003

43) Zeiler, S. R., & Krakauer, J. W. (2013). The interaction between training and plasticity in the post-stroke brain. *Current opinion in neurology*, *26*(6), 609. doi: 10.1097/WCO.0000000000000025

For peer review only - http://bmjopen.bmj.com/site/about/guidelines.xhtml

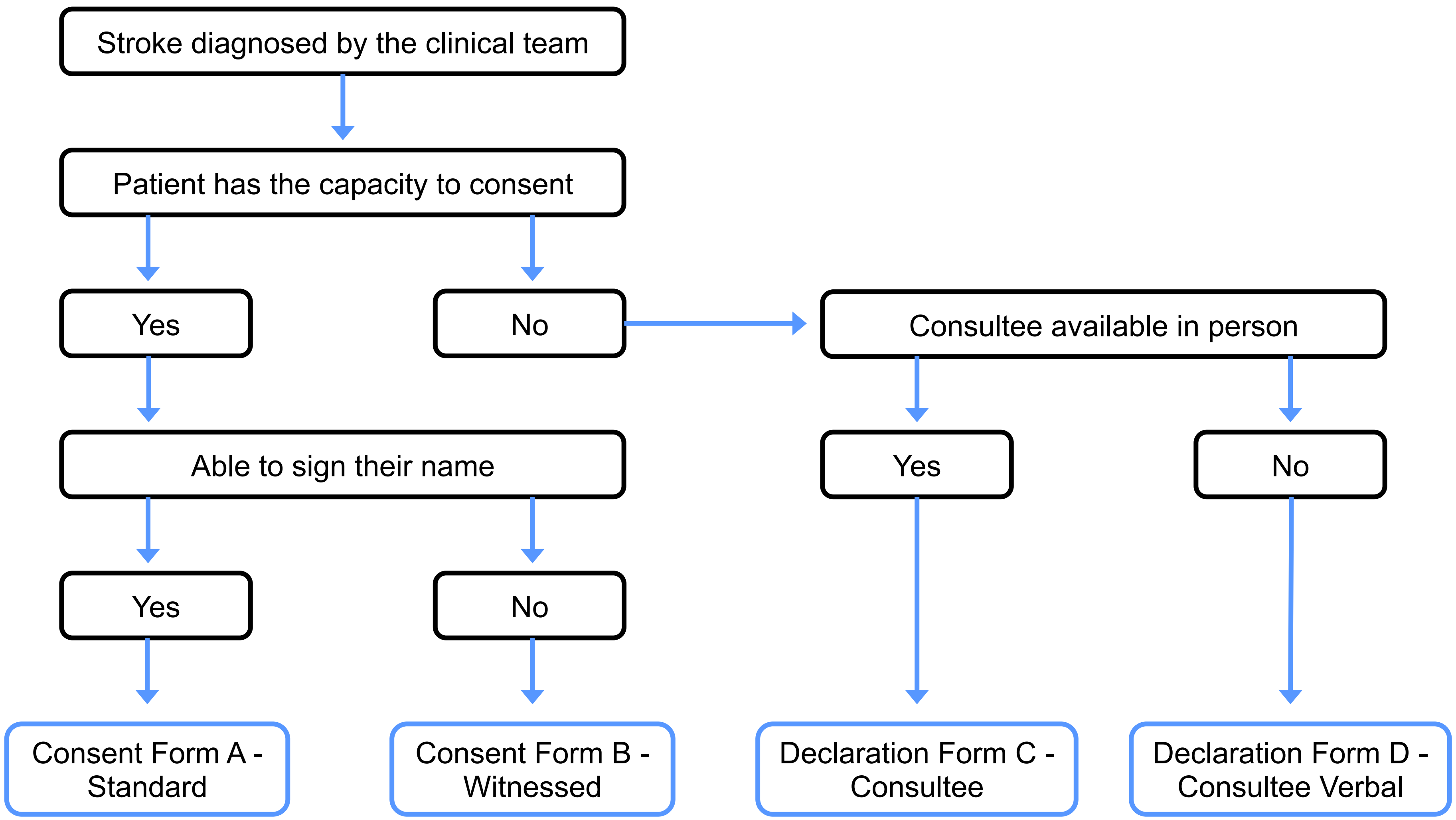
Stroke diagnosed by the clinical team
Patient has the capacity to consent
Yes
No
Consultee available in person
Able to sign their name
Yes
No
Yes
No
Consent Form A - Standard
Consent Form B - Witnessed
Declaration Form C - Consultee
Declaration Form D - Consultee Verbal

Participant Eligibility

Inclusion Criteria

- Aged 18 years or above.
- Stroke affecting the upper limb, as confirmed by clinical diagnosis.
- The participant is willing and able to give informed consent for participation in the study or a positive opinion from a consultee is provided by a family member or carer (relative or friend) willing to provide personal consultee (PC) advice.
- Recruited within 7 days of stroke.

Exclusion Criteria

- Diagnosis of other neurological condition affecting movement (such as Parkinson's disease, Multiple Sclerosis, etc).
- *Additional exclusion criteria apply to the application of transcranial magnetic stimulation (TMS), which may be required as part of the baseline assessment (PREP2 algorithm). Not all participants will require TMS (see Trial Design), and ineligibility will not affect participation.
    - Exclusion criteria for TMS procedure include: epilepsy/family history of epilepsy, seizures, metallic implants (above the shoulders), implanted electronics (e.g., pacemaker, cochlear implant, medical pump etc.), history of skull fracture or traumatic head injury, history of brain surgery, pregnancy, history of recurring headaches.

For peer review only - http://bmjopen.bmj.com/site/about/guidelines.xhtml

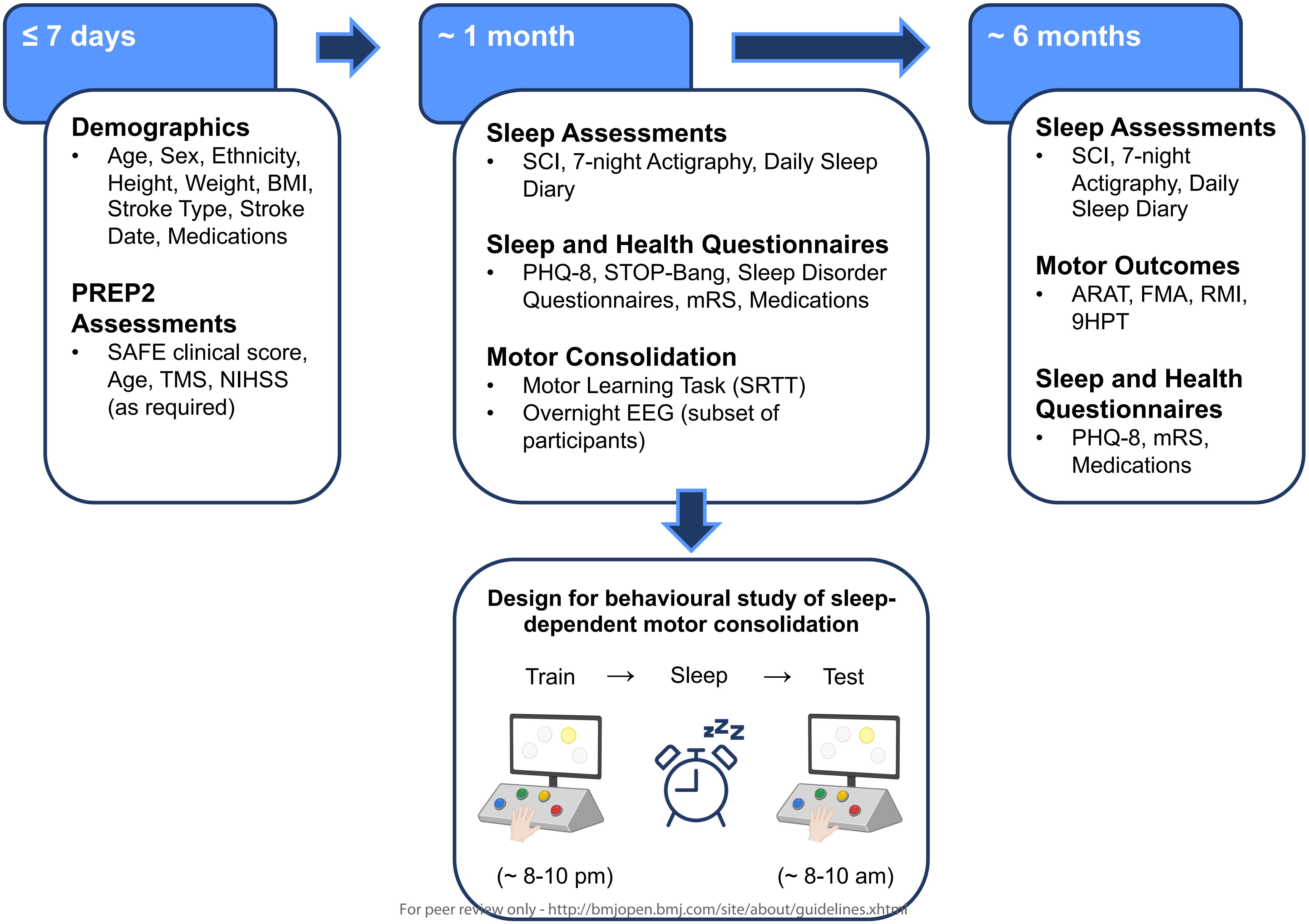
≤ 7 days
Demographics
Age, Sex, Ethnicity, Height, Weight, BMI, Stroke Type, Stroke Date, Medications
PREP2 Assessments
SAFE clinical score, Age, TMS, NIHSS (as required)
~ 1 month
Sleep Assessments
SCI, 7-night Actigraphy, Daily Sleep Diary
Sleep and Health Questionnaires
PHQ-8, STOP-Bang, Sleep Disorder Questionnaires, mRS, Medications
Motor Consolidation
Motor Learning Task (SRTT)
Overnight EEG (subset of participants)
~ 6 months
Sleep Assessments
SCI, 7-night Actigraphy, Daily Sleep Diary
Motor Outcomes
ARAT, FMA, RMI, 9HPT
Sleep and Health Questionnaires
PHQ-8, mRS, Medications
Design for behavioural study of sleep-dependent motor consolidation
Train → Sleep → Test
zZz
(~ 8-10 pm)
(~ 8-10 am)

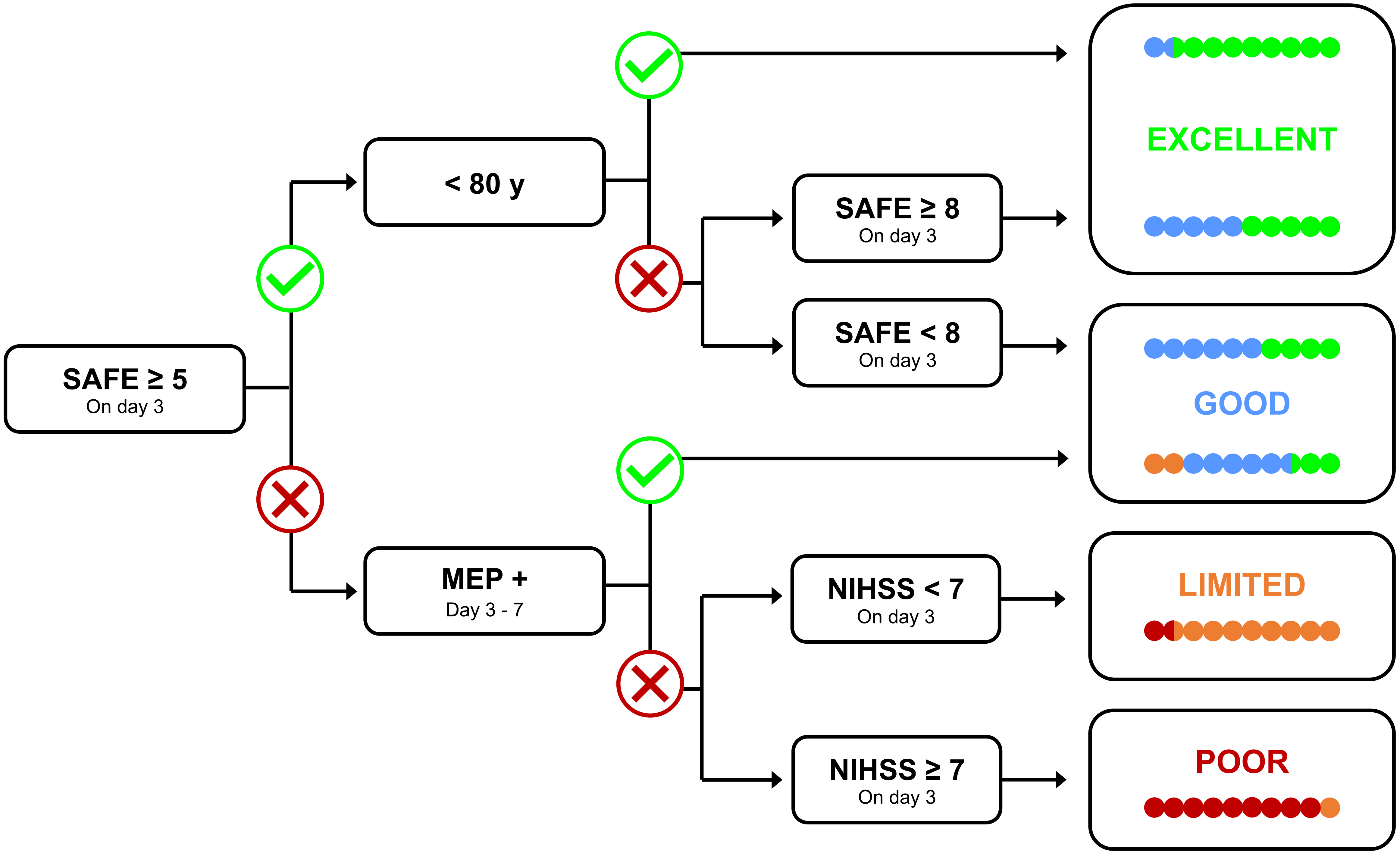
SAFE ≥ 5
On day 3
< 80 y
SAFE ≥ 8
On day 3
SAFE < 8
On day 3
MEP +
Day 3 - 7
NIHSS < 7
On day 3
NIHSS ≥ 7
On day 3
EXCELLENT
GOOD
LIMITED
POOR

Supplementary File 1: Button box technical note

Overview

The button box was designed with a button layout based on the specifications given by Gudberg, Wulf & Johansen-Berg[1] using commercially available parts.

Following initial evaluation, the design was revised to reduce button noise to minimise any potential for interference.

The button box communicates with the experiment software via a programmable USB-Human Interface Device (HID) module. This allows the buttons to be presented as any keyboard or joystick button required by the experiment software without the need for any additional software or drivers and is compatible with all modern operating systems.

Construction

The button box is built around an appropriately sized aluminium instrument case (CamdenBoss RTM2703-BR/B, RS stock no. 170-7825) with a sloped front on which four 60mm buttons (low-profile domed arcade buttons, purchased from Arcade World UK) are mounted according to the layout described in Gudberg et al.[1] such that they can be easily operated by the participant while resting their elbow on a flat surface, if needed (Figure 1). The buttons are connected to a programmable USB HID interface board (Ultimarc U-HID Nano) which is wrapped with heatshrink to provide insulation and mechanical protection and mounted internally to the box. The module's USB interface is then connected to a panel mount USB B socket (RS stock no. 874-1248) on the rear of the case via a short USB cable secured in place with heatshrink.

Noise reduction

The following steps were taken to minimise acoustic noise from the buttons:

The case was lined with sound deadening material (Teroson Adhesive bitumen acoustic insulation, RS stock no. 369-4715) in order to reduce the transmission of acoustic noise via the case.

The stock microswitches provided with the buttons were replaced with a quieter type (Cherry D44X, purchased from Arcade World UK) to reduce noise caused by switch actuation.

Thin self-adhesive foam was added to the back of the button plungers to reduce noise caused by impact of the plunger against the button housing.

For peer review only

A layer of the same sound deadening material used to line the case was applied to the back of the light diffuser and the opposing surface inside the button plunger to deaden sound conducted through the plunger.

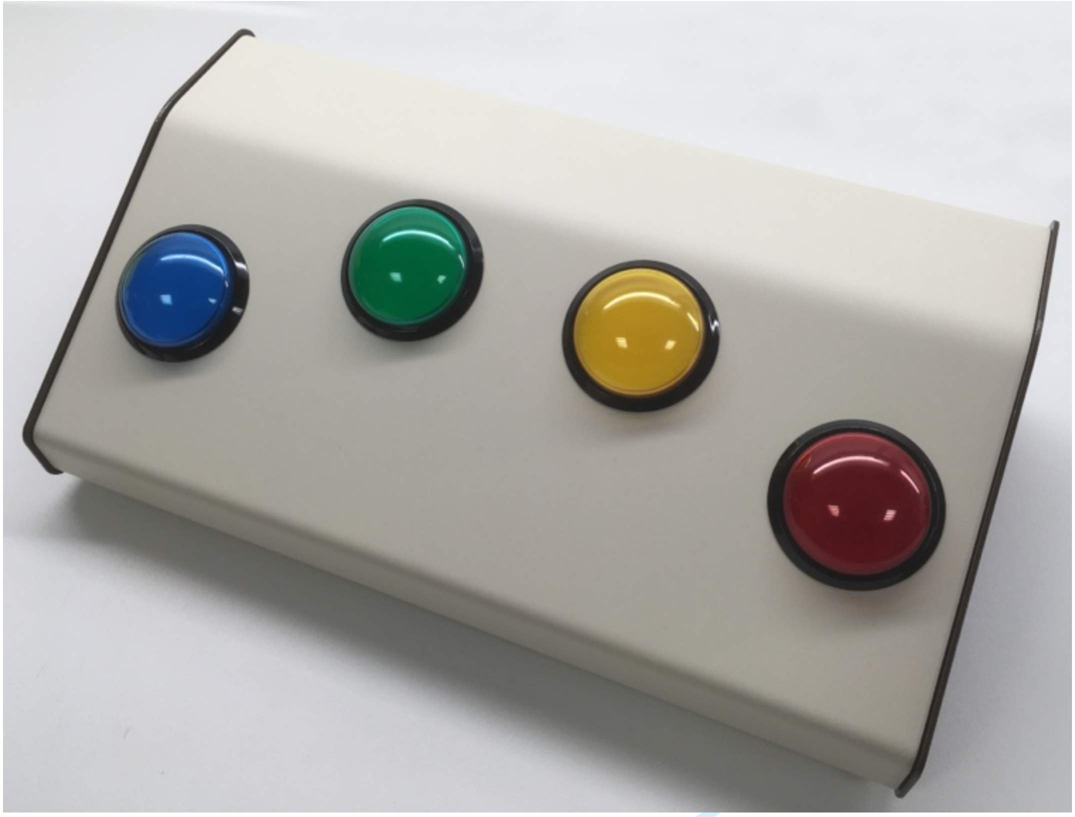

Figure 1: Image of the button box

References

Gudberg, C., Wulff, K., & Johansen-Berg, H. (2015). Sleep-dependent motor memory consolidation in older adults depends on task demands. Neurobiology of aging, 36(3), 1409–1416. doi: https://doi.org/10.1016/j.neurobiolaging.2014.12.014

Sleep and motor learning in stroke (SMiLES) - a longitudinal study investigating sleep-dependent consolidation of motor sequence learning in the context of recovery after stroke

Journal:	*BMJ Open*
Manuscript ID	bmjopen-2023-077442.R1
Article Type:	Protocol
Date Submitted by the Author:	04-Jan-2024
Complete List of Authors:	Weightman, Matthew; University of Oxford, Wellcome Centre for Integrative Neuroimaging (WIN), Nuffield Department of Clinical Neurosciences<br>Robinson, Barbara; University of Oxford, Wellcome Centre for Integrative Neuroimaging (WIN), Nuffield Department of Clinical Neurosciences<br>Mitchell, Morgan ; University of Oxford, Wellcome Centre for Integrative Neuroimaging (WIN), Nuffield Department of Clinical Neurosciences<br>Garratt, Emma; Buckinghamshire Oxfordshire and Berkshire West Integrated Care Board<br>Teal, Rachel; Oxford University Hospitals NHS Foundation Trust, MRC Stroke Unit, Oxford Centre for Enablement<br>Rudgewick-Brown, Andrew; University of Oxford, Wellcome Centre for Integrative Neuroimaging (WIN), Nuffield Department of Clinical Neurosciences<br>Demeyere, Nele; Oxford University, Wolfson Centre for the Prevention of Stroke and Dementia, Nuffield Department of Clinical Neurosciences<br>Fleming, Melanie K. ; University of Oxford, Wellcome Centre for Integrative Neuroimaging (WIN), Nuffield Department of Clinical Neurosciences<br>Johansen-Berg, Heidi; University of Oxford, Wellcome Centre for Integrative Neuroimaging (WIN), Nuffield Department of Clinical Neurosciences
**Primary Subject Heading**:	Rehabilitation medicine
Secondary Subject Heading:	Neurology
Keywords:	Stroke < NEUROLOGY, REHABILITATION MEDICINE, NEUROPHYSIOLOGY

SCHOLARONE™ Manuscripts

Sleep and motor learning in stroke (SMiLES) - a longitudinal study investigating sleep-dependent consolidation of motor sequence learning in the context of recovery after stroke

Authors: Matthew Weightman[1], Barbara Robinson[1], Morgan P Mitchell[1], Emma Garratt[2], Rachel Teal[3], Andrew Rudgewick-Brown[1], Nele Demeyere[4], Melanie K Fleming[1^], Heidi Johansen-Berg[1^]

^ These authors contributed equally

Affiliations:

[1]Wellcome Centre for Integrative Neuroimaging (WIN), Nuffield Department of Clinical Neurosciences, University of Oxford, Oxford, OX3 9DU, UK

[2]Buckinghamshire, Oxfordshire and Berkshire West Integrated Care Board (BOB ICB), Oxford OX4 6LB

[3]MRC Stroke Unit, Oxford Centre for Enablement, Oxford University Hospitals NHS Foundation Trust, Oxford, United Kingdom, OX3 7HE

[4]Wolfson Centre for the Prevention of Stroke and Dementia, Nuffield Department of Clinical Neurosciences, University of Oxford, Oxford, OX3 9DU

For peer review only

Corresponding Author:
Matthew Weightman
Wellcome Centre for Integrative Neuroimaging (WIN),
Nuffield Department of Clinical Neurosciences,
University of Oxford,
Oxford,
OX3 9DU

matthew.weightman@ndcn.ox.ac.uk

Word Count: 4142

Keywords: *Stroke, Rehabilitation, Motor Consolidation, Sleep*

For peer review only

Abstract

Introduction: There is growing evidence that sleep is disrupted after stroke, with worse sleep relating to poorer motor outcomes. It is also widely acknowledged that consolidation of motor learning, a critical component of post-stroke recovery, is sleep-dependent. However, whether the relationship between disrupted sleep and poor outcomes after stroke is related to direct interference of sleep-dependent motor consolidation processes, is currently unknown. Therefore, the aim of the present study is to understand whether measures of motor consolidation mediate the relationship between sleep and clinical motor outcomes post-stroke.

Methods and Analysis: We will conduct a longitudinal observational study of up to 150 participants diagnosed with stroke affecting the upper limb. Participants will be recruited and assessed within 7 days of their stroke and followed up at approximately 1 and 6 months. The primary objective of the study is to determine whether sleep in the sub-acute phase of recovery explains the variability in upper limb motor outcomes after stroke (over and above predicted recovery potential from the PREP2 algorithm) and whether this relationship is dependent on consolidation of motor learning. We will also test whether motor consolidation mediates the relationship between sleep and whole-body clinical motor outcomes, whether motor consolidation is associated with specific electrophysiological sleep signals, and sleep alterations during sub-acute recovery.

Ethics and Dissemination: This trial has received both Health Research Authority (HRA), Health and Care Research Wales (HCRW), and National Research Ethics Service approval (IRAS: 304135; REC: 22/LO/0353). The results of this trial will help to enhance our understanding of the role of sleep in recovery of motor function after stroke and will be disseminated via presentations at scientific conferences, peer-

For peer review only

reviewed publication, public engagement events, stakeholder organisations, and other forms of media where appropriate.

Trial Registration: ClinicalTrials.gov: NCT05746260**,** registered on 27th February 2023.

Strengths and Limitations

- Longitudinal study, with early assessment (≤ 7 days post-stroke) and follow-ups throughout sub-acute recovery.
- All assessments carried out where the patient is currently residing to reduce burden.
- Inclusion of patients without capacity to consent to increase the diversity of study sample.
- Some patients may not be able to complete all aspects of the study due to level of impairment.
- Likely between-subject variability in the amount of upper extremity therapy/treatment received during study enrolment.

For peer review only

Introduction

Stroke is a leading cause of worldwide disability, with over 50% of stroke survivors experiencing long-term motor impairment (1-3). It is widely believed that good recovery of movement after stroke is dependent on motor learning (4). Indeed, most current rehabilitative practises aiming to improve motor function after stroke place a key focus on learning new, or relearning old, ways in which to successfully carry out activities of daily living (5,6). Motor learning is thought to be especially important for post-stroke recovery due to its capacity to drive structural and functional changes in the brain, termed neuroplasticity (7,8) As good rehabilitation outcomes after stroke are dependent on the ability of the brain to learn and reorganise, it is critical we understand how neuroplasticity occurs and how it can be influenced in the context of recovery.

A key element of our understanding of neuroplasticity – which has yet to be fully explored in the context of stroke rehabilitation - is consolidation of memories during sleep (4) Improvements in motor learning are known to not only occur online (during practise itself) but also offline, during periods of rest (9). For some time now, sleep has been proposed to be actively engaged in the offline consolidation of motor memories (10-12). The reactivation of task-related neural activity during sleep is believed to underpin offline memory processing, by transforming labile memory traces into persistent representations, readily available for retrieval (13,14). These reactivations are also closely linked to fine-tuned interactions between electrophysiological signatures of slow wave sleep, namely; neocortical slow oscillations and thalamocortical sleep spindles, known to be key correlates of memory consolidation (15-18). Taken together, this evidence highlights the importance of sleep to the offline consolidation of motor memories.

For peer review only - http://bmjopen.bmj.com/site/about/guidelines.xhtml

Notwithstanding, sleep disruption is commonplace after stroke, with more fragmented sleep, reduced sleep efficiency, and less total sleep time commonly reported (19-22). Evidence suggests that poor sleep after brain injury, including stroke, is often associated with worse motor/cognitive impairments, quality of life, and mood (22-24). Furthermore, we and others have demonstrated that sleep disruption during rehabilitation after stroke and/or brain injury is associated with slower functional recovery and worse motor outcomes (23,25). However, whether this relationship exists because impaired sleep post-stroke directly disrupts memory consolidation processes, or reflects other factors that influence sleep and clinical outcomes e.g., depression, motivation etc. is yet unknown. Given that clinical gains during therapy are likely to depend on both improvements during training and sleep, it is reasonable to suggest that poor motor outcomes may be, at least in part, due to disrupted sleep and thereby associated memory processes.

Trial Objectives

Considering the link between sleep disruption and recovery, the aim of the present study is to understand to what extent behavioural measures of motor consolidation mediate the relationship between sleep quality and clinical motor outcomes post-stroke. We will investigate whether sleep measures in the sub-acute phase explain variation in motor outcomes (over and above variation explained by baseline assessment, and other covariates) and whether this relationship is mediated by variation in behavioural measures of overnight consolidation. More specifically, we aim to:

Primary Objectives

For peer review only - http://bmjopen.bmj.com/site/about/guidelines.xhtml

1) Test whether sleep measures at ~1-month post-stroke explain variability in recovery of movement of the paretic upper limb at ~6 months.
2) Assess whether the relationship between sleep and recovery after stroke depends on motor consolidation.

Secondary Objective

1) Determine if motor consolidation at ~1-month post-stroke mediates the relationship between sleep and clinical outcomes, such as whole-body motor impairment, mobility, and hand function of the more-affected side, measured at ~6 months post-stroke.

Exploratory Objectives

1) Explore whether motor consolidation is associated with specific electrophysiological markers of sleep after stroke.
2) Investigate changes in sleep alongside recovery over the first 6 months after stroke.
3) Explore correlations between sleep disruption and motor function between ~1- and 6-months post-stroke.

Methods and Analysis

Participant Recruitment

Up to 150 participants, diagnosed with stroke affecting the upper limb, will be recruited. All participants will be identified from stroke wards at the acute stage post stroke (≤ 7 days). Patients will be approached by a member of the clinical team or a clinical research facilitator (e.g., research nurse) to determine potential interest and provide information about the study. Upon verbal consent (or in case of expressive speech problems, a clear non-verbal indication e.g., a nod), a researcher will

For peer review only

approach interested patients to go through the informed consent process for the study. It will be made clear that participation is voluntary and will not affect their medical or rehabilitative care. Where the mental capacity to provide informed consent is in doubt, a mental capacity assessment for consenting to the research study will be conducted and appropriate consultee advice will be sought from a familial carer, other relative, or close friend if appropriate (Figure 1). Inclusion and exclusion criteria are shown below. The trial was registered on February 27th, 2023 (prior to recruitment of the first participant; NCT05746260), opened to recruitment in March 2023, with a planned trial end date of March 2027.

[Insert Figure 1]

Figure 1: *Consent process, including capacity to consent decision framework.*

<u>Sample Size Calculations</u>

Based on findings from previous work in inpatients with brain injury (23), we predict that sleep measures increase variance in motor outcomes explained by ~10%. Therefore, to achieve a power of 0.9 (1-β error probability) with a significance level (alpha) of < 0.05, a total sample size of ~n = 100 is recommended (calculated using G*Power; linear multiple regression, with a fixed model). In anticipation of participant attrition, we will aim to recruit 150 participants.

<u>Inclusion Criteria</u>

- Aged 18 years or above.
- Stroke affecting the upper limb, as confirmed by clinical diagnosis (no specific requirement regarding affected side and limb dominance).

For peer review only

For peer review only - http://bmjopen.bmj.com/site/about/guidelines.xhtml

- The participant is willing and able to give informed consent for participation in the study or a positive opinion from a consultee is provided by a family member or carer (relative or friend) willing to provide personal consultee (PC) advice.
- Recruited ≤ 7 days of stroke.

Exclusion Criteria

- Diagnosis of other neurological condition affecting movement (such as Parkinson's disease, Multiple Sclerosis, etc).
- *Additional exclusion criteria apply to the application of transcranial magnetic stimulation (TMS), which may be required as part of the baseline assessment (PREP2 algorithm). Not all participants will require TMS (see Trial Design), and ineligibility will not affect participation.
  - Exclusion criteria for TMS procedure include: epilepsy/family history of epilepsy, seizures, metallic implants (above the shoulders), implanted electronics (e.g., pacemaker, cochlear implant, medical pump etc.), history of skull fracture or traumatic head injury, history of brain surgery, pregnancy, history of recurring headaches.

Trial Design

The trial will be conducted as a longitudinal observational study. The direct clinical care team or research nurse will identify and screen potential participants, checking their medical history to confirm eligibility. Should participants be interested and meet all eligibility criteria, a researcher will then arrange a consultation for the baseline visit (≤ 7 days post-stroke), where they will go over the details of the study, answer any remaining questions and complete the informed consent process (Figure 1).

For peer review only - http://bmjopen.bmj.com/site/about/guidelines.xhtml

Baseline assessment will be conducted as soon as possible thereafter. Following consent, participants will be enrolled in the trial and assigned a unique study code, and the direct care team will be informed (verbally) that the patient has consented to take part in the study. We will also inform each participant's GP of consent in the study.

[Insert Figure 2]

Figure 2: *Flowchart of the trial timeline and assessments. BMI, Body Mass Index; SAFE, Shoulder Abduction Finger Extension; NIHSS, National Institute of Health Stroke Scale; SCI, Sleep Condition Indicator; PHQ-8, Patient Health Questionnaire; STOP-Bang, Obstructive Sleep Apnoea Questionnaire; SRTT, Serial Reaction Time Task; EEG, Electroencephalography; ARAT, Action Research Arm Test; FMA, Fugl-Meyer Assessment; RMI, Rivermead Mobility Index; BBT, Box and Blocks Test; mRS, modified Rankin Scale.*

For peer review only

Outcome Measures

Our main outcomes will be used to determine whether sleep in the sub-acute phase of recovery after stroke explains the variability seen in upper limb motor outcomes and whether this relationship is dependent on overnight consolidation of motor memories. The main outcome measures are described in detail in the descriptions of assessment sessions below. Briefly:

Sleep disruption will be measured via 7-night actigraphy recordings and sleep diaries, indicating estimated total sleep time, wakefulness after sleep onset (WASO), and sleep fragmentation.

Symptoms of insomnia will be recorded using the Sleep Condition Indicator questionnaire (SCI) (26,27), with higher scores indicating fewer symptoms of insomnia (range 0-32).

Motor consolidation will be measured at approximately 1-month after stroke and will describe the change in performance (response time/accuracy) from evening training to morning retest in the adapted Serial Reaction Time Task (SRTT) (12,28).

Upper limb function will be assessed at approximately 6 months post-stroke using the Action Research Arm Test (29). Scores range between 0-57, with higher scores indicates better upper limb ability.

Secondary outcome measures include: whole body motor impairment, hand dexterity, and mobility at ~ 6 months after stroke, assessed via the Fugl-Meyer Assessment (FMA) (30), Nine Hole Peg Test (9HPT) (31) and Rivermead Mobility Index (RMI) (32), respectively (higher scores across all measurements indicates better movement/performance). These outcomes will help to determine whether motor consolidation mediates the relationship between sleep and different aspects of motor recovery. Actigraphy derived sleep measures and SCI scores at 1- and 6-months post-stroke will be used to explore changes in sleep quality during sub-acute recovery. Additionally, electroencephalogram (EEG) derived sleep measures (e.g., slow oscillation amplitude, sleep spindle density, slow oscillation-sleep spindle coupling etc) collected in a sub-set of participants at 1-month post-stroke will be used to discern whether consolidation of motor learning is associated with specific sleep signals in this population.

Other pre-specified measures include: symptoms of depression via the patient health questionnaire (PHQ-8) (33), stroke severity/disability via the modified Ranking Scale (mRS), and physical activity (actigraphy recordings) assessed at approximately 1 and 6 months post-stroke, in addition to the risk of obstructive sleep apnoea (OSA) using the STOP-Bang questionnaire (34) and presence of sleep

For peer review only

disorders (35) at 1-month post-stroke. These will be used as covariates in analyses as appropriate.

Baseline Assessment

The baseline assessment will take place ≤ 7 days post-stroke. We will record participant demographics, including: age, sex, ethnicity, weight, height, stroke type, stroke date, and current medications (Figure 2).

To characterise expected upper limb recovery potential, we will follow procedures for the Predict Recovery Potential (PREP2) algorithm (36). In the initial days post-stroke, the PREP2 algorithm combines neurological biomarkers and clinical measures, to predict upper limb functional outcomes at 3 months (Figure 3). Patients can be categorised into one of four different recovery categories: excellent, good, poor, and limited, with predictions shown to be accurate for up to 80% of patients (36,37). A shoulder abduction and finger extension (SAFE) score will be calculated using the Medical Research Council (MRC) grades to determine participants' strength in each of these movements. Movements are scored between 0 and 5, where 0 represents no palpable muscle activity and 5 describes normal strength and range of movement. Participants with a SAFE score of < 5 will require transcranial magnetic stimulation (TMS) to establish the presence of motor evoked potentials (MEPs) in the paretic upper limb for further categorisation. Participants will be screened separately for TMS safety and participants can still take part in the study if unable to undergo TMS. If a participant requires TMS - and is deemed appropriate to do so as per the screening checklist - we will use surface electromyography (EMG) to record muscle activity from the extensor carpi radialis (ECR) and first dorsal interosseus (FDI) of the affected upper limb between 3-7 days after stroke. A patient

For peer review only

will be classified as MEP+ if MEPs of any amplitude are elicited with a consistent latency in response to at least 5 stimuli at rest or during voluntary muscle facilitation and MEP- if no MEPs can be elicited at 100% maximum stimulator output (MSO) either at rest or while attempting voluntary muscle facilitation. We estimate that approximately 30% of participants will require TMS (36). If a participant is unable to undergo TMS, we will use all other biomarker information available and categorise on a 'worst case scenario' basis (analyses will be conducted with and without these participants included to ensure that this estimation does not influence findings). Age and National Institute for Health Stroke Scale (NIHSS) scores will also be collected to dictate final categorisation.

[Insert Figure 3]

Figure 3: *PREP2 algorithm, included with permission, under license CC BY-NC-ND 4.0 (https://presto.auckland.ac.nz) (36). The algorithm uses neurological and clinical biomarkers to predict four different categories of potential recovery outcomes at 3 months after stroke. SAFE, Shoulder Abduction Finger Extension score; MEP, Motor Evoked Potential; NIHSS, National Institute of Health Stroke Scale.*

1-month post-stroke

All procedures involved in the 1-month visit will be conducted at the location the patient is currently residing, with researchers visiting to collect data and set up equipment. At ~1-month after stroke (no more than 12 weeks), sleep measures will be collected via 7-night actigraphy recordings (measuring day/night activity, estimated total sleep time, WASO, and sleep fragmentation index) and completion of the SCI) (26). For actigraphy measures, participants will wear a waterproof monitor on their least affected wrist (MotionWatch 8, CamNTech). A sleep diary, detailing

For peer review only

approximately what time participants fell asleep and woke up for each of the 7 nights, will also be completed (Figure 2). We have previously, and continue to, successfully implement similar actigraphy and diary-based approaches in multiple research projects comprising of over 200 patients, proving to be safe and tolerable (22,23,38,39). We will assess the presence of other sleep disorders via completion of a sleep screening and STOP-Bang questionnaires, and mood by means of the PHQ-8. Additionally, we will ask participants to complete a series of questions to determine the score for the mRS, and record current medications. We will record where the participant is residing at the time of the visit (e.g., home, rehabilitation unit, care home etc.), and ask participants to self-report whether they have received motor rehabilitation and/or sleep therapy following their stroke, and if so, approximately how much (e.g., sessions per week, average session length, and upper and/or lower limb focus).

We will assess consolidation of motor sequence learning using an adapted version of the SRTT, see Figure 2. The task has been adapted so that participants are required to move their entire upper limb and press each button with their whole hand rather than individual fingers as is customary in SRTTs (12), see supplementary file 1. Studies using similar tasks have reported effective learning in stroke patients using the paretic upper limb (40) and sleep-dependent motor improvements in older adult participants (12). Participants will learn to press buttons on a button box with their affected limb following a repeated 8-item sequence. On-screen representations of each button will become highlighted, indicating which button to press. Once a response is given the next target cue in the sequence will become highlighted, and so on, regardless of whether the correct response is given. Participants will perform two different versions of the task (training and test), one in the evening and then one

For peer review only

the following morning, respectively (Figure 2). The training task will include 20 blocks of trials, with three repetitions of the 8-item sequence in each block. The test, aimed to determine sleep-dependent changes, will only include 3 blocks of trials to avoid confounds related to additional learning. To probe sequence specific learning, two blocks of 'random' sequences will be inserted at the beginning and towards the end of each the training and test tasks. We will not explicitly inform participants of the sequence structure, however, a banner will appear at the top of the screen during every block, indicating whether the button presses will follow the sequence or a random order. At the end of the test task, participants will be asked to attempt to replicate as much of the sequence order as they can remember/were aware of without being shown the cues. Participants will be asked to train on the task between 8-10 pm or approximately 30-60 minutes before they intend to go to sleep and then be tested the following morning between 8-10am or at least 30 minutes after wake. The training will take approximately 20-30 minutes and retest will take no more than 10 minutes. Measures of both speed (response times) and accuracy will be recorded.

In a subset of participants, we will also measure overnight brain activity using a portable low-density EEG device. Evidence suggests that available products are efficacious for monitoring sleep-related electrophysiological signals and are comparable to gold-standard polysomnography (PSG) protocols (41,42). We have also previously explored the feasibility of portable at-home EEG device use by stroke survivors at home (unpublished data), with no major issues reported. Participants will be asked to wear the EEG device for three consecutive nights: one acclimatisation night followed by the night prior to completing the motor learning task, and the night of the motor learning task. Only participants who are residing at home will be invited

For peer review only

For peer review only - http://bmjopen.bmj.com/site/about/guidelines.xhtml

for this assessment and they may opt out with no consequence to any of the other aspects of the study.

6 months post-stroke

We will assess sleep using the same protocol as detailed for the 1-month visit (SCI, 7-night actigraphy, and daily sleep diary). Similarly, we will measure mood (PHQ-8), disability (mRS), and record current medications (Figure 2). We will also document where the participant is residing, and self-report of whether they have received motor rehabilitation and/or sleep therapy (and if so, how much) since the previous assessment.

To determine functional recovery 6 months after stroke, we will carry out a number of standardised clinical assessments requiring participants to make everyday movements with their hands, arms, and legs. These include the ARAT, upper and lower extremity sections of the FMA, the RMI and the 9HPT. Again, researchers will visit participants in their homes or where they are currently residing to carry out all aspects of the 6-month assessment. In instances where participants prefer not to complete the movement assessments at their residing location, they will be invited to attend the research lab to complete these.

Data Management

Direct access to data will be granted to authorised representatives from the trial sponsor and host institution for monitoring and/or audit of the study in compliance with regulations. Participants will be assigned a unique study code, which will be used on all study documents (apart from consent forms) and all documents will be stored safely in confidential conditions. Anonymous data will be shared openly

For peer review only - http://bmjopen.bmj.com/site/about/guidelines.xhtml

through a data sharing initiative (e.g., Open Science Framework; OSF) following publication or public release of the study results.

Data/Statistical Analyses

Primary Analyses

To test whether sleep in the sub-acute phase of recovery after stroke explains variability seen in upper limb motor outcomes, we will use the general linear model to test the association between sleep measures at 1 month and ARAT score at 6-months post-stroke. We will include relevant covariates such as age, side affected, anticipated recovery outcome category (from PREP2 algorithm), PHQ-8 score, likely presence of sleep disorders, and the activity metric from actigraphy recordings, etc. as appropriate. To determine whether this relationship depends on motor consolidation, we will test for a mediation effect of motor consolidation at 1 month on this association. Motor consolidation will be determined by calculating the difference in performance from the end of evening training relative to the morning retest, with random trial blocks used to evaluate any sequence specific effects.

Secondary Analyses

To test whether motor consolidation mediates the relationship between sleep and a broader range of clinical outcomes and timepoints, we will use the general linear model, testing for a mediation effect of motor consolidation at 1-month post-stroke on the correlation between sleep measures at 1-month and ARAT, FMA, 9HPT, and RMI scores at 6 months post-stroke.

For peer review only

For peer review only - http://bmjopen.bmj.com/site/about/guidelines.xhtml

Exploratory Analyses

To explore whether motor consolidation is associated with specific sleep signals after stroke, we will use the general linear model to correlate motor consolidation with EEG derived sleep metrics at 1-month post-stroke. Finally, to explore changes in sleep alongside recovery we will use the general linear model to test for changes in sleep measures from 1-month to 6 months post-stroke.

Study Monitoring

The study management group, consisting of the principal investigator and members of the research team, will meet multiple times per year to monitor the progress of the study. Participants will be regularly monitored, and data evaluated to check for protocol compliance and accuracy in relation to source documents. Responsible personnel from the NHS Trust(s) and/or sponsor may also monitor or audit the trial where appropriate.

Throughout the trial timeline, we will check with participants for the occurrence of any adverse events (AEs). Potential expected minor AEs include distress associated with completing mood questionnaires and skin irritation from wearing the actigraphy monitor. Any AEs that do occur during study enrolment will be documented and participants will be advised on the most appropriate course of action, e.g., withdrawing from particular assessments or contacting their General Practitioner (GP). There is a potential, but very small, risk of seizure with TMS. The most recent update to safety guidelines suggested that, given the large number of patients who have received TMS and the small number of seizures, the risk of TMS to induce seizures is very low (43). All participants who require TMS will be screened prior to testing and all procedures will be conducted within the safety guidelines. There are

For peer review only

no anticipated serious AEs (SAEs) associated with the present trial. Any SAEs that occur (and deemed unexpected and related to research procedures) will be reported to the appropriate ethics committee within 15 days of awareness of the event, using the Health Research Authority (HRA) report of SAE form (see HRA website for further info).

During the course of the trial a participant may choose to withdraw at any time point and for any reason. The reason for withdrawal will be recorded if known, but participants are not obliged to provide a reason. Data from withdrawn participants may be kept and used for analyses as appropriate. If there is sufficient time to conduct all necessary study assessments within the study period, additional participants will be recruited to replace withdrawals. If a participant is unable to comply with one aspect of the assessment (e.g., the motor learning task), then they may continue other assessments unless they choose to withdraw entirely. Moreover, an investigator may choose to discontinue a participant from the trial if it is considered necessary for any reason e.g., significant protocol deviation, non-compliance, ineligibility (either arising during the study period or retrospectively having been overlooked at screening) etc.

For peer review only

Patient and Public Involvement

Stroke survivors were involved in producing and reviewing all participant facing documents. We also encouraged patients to give feedback on the motor learning task at different public/patient engagement events throughout the design of the study and during pilot testing. This feedback was used to write instructions and edit task difficulty. We will seek further assistance from patient/public contributors when

For peer review only - http://bmjopen.bmj.com/site/about/guidelines.xhtml

disseminating results from the trial - in the form of lay summaries - to ensure key messages are communicated in a meaningful way to stroke communities.

Ethics and Dissemination

This trial has received both Health Research Authority (HRA), Health and Care Research Wales (HCRW), and National Research Ethics Service approval (IRAS: 304135; REC: 22/LO/0353). The study is registered on a publicly accessible database (clinicaltrials.gov: NCT05746260). Results of the study will be uploaded to clinicaltrials.gov within 12 months of the end of trial and disseminated via presentations at scientific conferences, peer-reviewed publication, public engagement events, stakeholder organisations, and other forms of media where appropriate.

For peer review only

Limitations

Despite efforts to develop a robust study design, there remain some limitations to the present protocol. Firstly, due to logistical constraints, consolidation of motor learning is only assessed at one timepoint, approximately 1-month post-stroke. We are working on the assumption that consolidation on any given night will be determined by a combination of *stable factors* (such as habitual sleep quality) as well as factors *specific to that night* (such as whether someone's sleep was disturbed by external noises). Our study is designed to capture the former and not the latter. To capture the former, measuring consolidation on any night can provide an estimate of habitual consolidation performance. To capture the latter would require assessments of consolidation on multiple nights following rehabilitation sessions, which would be too complex to administer and too burdensome for participants. Secondly, the accuracy

of sleep measurements may be limited due to being derived mainly from actigraphy recordings (e.g., movement related), as well as EEG recordings collected at only one timepoint post-stroke for a subset of participants. Despite this, actigraphy-derived sleep measures have been shown to relate to motor outcomes over periods of rehabilitation (23). Finally, the self-report nature of some key outcomes e.g., SCI may limit outcome sensitivity.

Summary and Conclusions

The present trial aims to investigate the relationship between sleep, motor consolidation, and clinical outcomes after stroke. The importance of motor learning and neuroplasticity in post-stroke recovery is widely recognised (4,8,44), however, the role of sleep in offline consolidation of motor memories is an area that has yet to be fully explored in the context of stroke rehabilitation. We will investigate whether sleep measures in the sub-acute stage of stroke explain variability in recovery of upper limb function, assessing the relationship between sleep and recovery in terms of motor consolidation, and determining whether motor consolidation mediates the relationship between sleep and clinical outcomes. Using wearable remote technologies, we will also explore electrophysiological markers of sleep after stroke, changes in sleep alongside recovery, and correlations between sleep and motor function. We aim to build on our previous work showing that sleep is disrupted after stroke/brain injury and predicts poorer recovery (22,23), to determine whether the consolidation of motor memories is a candidate process to mediate this relationship. We hope the findings of this study will help to enhance our understanding of the role of sleep in stroke recovery and may inform the development of novel interventions for improving post-stroke rehabilitation outcomes.

For peer review only - http://bmjopen.bmj.com/site/about/guidelines.xhtml

Acknowledgements

We would like to thank the individuals at OSRU and NIHR CRN Stroke for their support in setting up, recruiting for, and facilitating the trial. Thanks also to Sebastian Rieger for his input regarding behavioural motor task hardware design.

Author Contributions

MW, BR, MKF, and HJB conceived and designed the protocol with input from MM. EG, RT and ND facilitate recruitment, consent process, and data collection. ARB designed and constructed the hardware for the behavioural motor task. MW and BR are involved in the day-to-day running of the trial. MW drafted the manuscript. All authors edited, revised and approved the final version of the manuscript.

Funding

This study is funded by the Wellcome Trust (Principal Research Fellowship to HJB; 222446/Z/21/Z) and supported by the NIHR Oxford (Oxford University Hospitals) and Oxford Health Biomedical Research Centres (BRC). These funding sources had no role in the design of this study and will not have any role during its execution, analyses, or interpretation of data. The Wellcome Centre for Integrative Neuroimaging is supported by core funding from the Wellcome Trust.

Conflicting Interests

No conflicting interests declared.

Rights Retention

For peer review only

This research is funded in whole, or in part, by the Wellcome Trust [Grant number 222446/Z/21/Z]. For the purpose of open access, the author has applied a CCBY public copyright license to any Author Accepted Manuscript version arising from this submission.

For peer review only

References

1) Wolfe, C. D. (2000). The impact of stroke. *British medical bulletin*, *56*(2), 275–286. doi: https://doi.org/10.1258/0007142001903120

2) Raghavan, P. (2015). Upper limb motor impairment after stroke. *Physical Medicine and Rehabilitation Clinics*, *26*(4), 599–610. doi: https://doi.org/10.1016/j.pmr.2015.06.008

3) Feigin, V. L., Brainin, M., Norrving, B., Martins, S., Sacco, R. L., Hacke, W., . . . Lindsay, P. (2022). World stroke organization (WSO): global stroke fact sheet 2022. *International Journal of Stroke*, *17*(1), 18–29. doi: https://doi.org/10.1177/17474930211065917

4) Krakauer, J. W. (2006). Motor learning: its relevance to stroke recovery and neurorehabilitation. Current opinion in neurology, 19(1), 84–90. doi: 10.1097/01.wco.0000200544.29915.cc

5) Winstein, C., Lewthwaite, R., Blanton, S. R., Wolf, L. B., & Wishart, L. (2014). Infusing motor learning research into neurorehabilitation practice: a historical perspective with case exemplar from the accelerated skill acquisition program. *Journal of neurologic physical therapy: JNPT*, *38*(3), 190. doi: 10.1097/NPT.0000000000000046

6) Leech, K. A., Roemmich, R. T., Gordon, J., Reisman, D. S., & Cherry-Allen, K. M. (2021). Updates in motor learning: Implications for physical therapist practice and education. Physical therapy. doi: https://doi.org/10.1093/ptj/pzab250

7) Dayan, E., & Cohen, L. G. (2011). Neuroplasticity subserving motor skill learning. *Neuron*, *72*(3), 443–454. doi: https://doi.org/10.1016/j.neuron.2011.10.008

8) Sampaio-Baptista, C., Sanders, Z.-B., & Johansen-Berg, H. (2018). Structural plasticity in adulthood with motor learning and stroke rehabilitation. *Annual review of neuroscience, 41, 25–40. doi: https://doi.org/10.1146/annurev-neuro-080317-062015*

9) Robertson, E. M., Pascual-Leone, A., & Miall, R. C. (2004). Current concepts in procedural consolidation. *Nature Reviews Neuroscience*, *5*(7), 576–582. doi: https://doi.org/10.1038/nrn1426

10) Walker, M. P., Brakefield, T., Seidman, J., Morgan, A., Hobson, J. A., & Stickgold, R. (2003). Sleep and the time course of motor skill learning. *Learning & memory*, *10*(4), 275–284. doi: 10.1101/lm.58503

11) Stickgold, R., & Walker, M. P. (2007). Sleep-dependent memory consolidation and reconsolidation. *Sleep medicine*, *8*(4), 331–343. doi: https://doi.org/10.1016/j.sleep.2007.03.011

For peer review only - http://bmjopen.bmj.com/site/about/guidelines.xhtml

12) Gudberg, C., Wulff, K., & Johansen-Berg, H. (2015). Sleep-dependent motor memory consolidation in older adults depends on task demands. Neurobiology of aging, 36(3), 1409–1416. doi: https://doi.org/10.1016/j.neurobiolaging.2014.12.014

13) Rasch, B., & Born, J. (2013). About sleep's role in memory. *Physiological reviews*. doi: https://doi.org/10.1152/physrev.00032.2012

14) Klinzing, J. G., Niethard, N., & Born, J. (2019). Mechanisms of systems memory consolidation during sleep. Nature neuroscience, 22(10), 1598–1610. doi: https://doi.org/10.1038/s41593-019-0467-3

15) Morin, A., Doyon, J., Dostie, V., Barakat, M., Tahar, A. H., Korman, M., ... Carrier, J. (2008). Motor sequence learning increases sleep spindles and fast frequencies in post-training sleep. *Sleep*, *31*(8), 1149–1156. doi: https://doi.org/10.5665/sleep/31.8.1149

16) Born, J., & Wilhelm, I. (2012). System consolidation of memory during sleep. *Psychological research*, *76*, 192–203. doi: https://doi.org/10.1007/s00426-011-0335-6

17) Ramanathan, D. S., Gulati, T., & Ganguly, K. (2015). Sleep-dependent reactivation of ensembles in motor cortex promotes skill consolidation. *PLoS biology*, *13*(9), e1002263. doi: https://doi.org/10.1371/journal.pbio.1002263

18) Fogel, S., Albouy, G., King, B. R., Lungu, O., Vien, C., Bore, A., . . . Doyon, J. (2017). Reactivation or transformation? Motor memory consolidation associated with cerebral activation time-locked to sleep spindles. *PloS one*, *12*(4), e0174755. doi: https://doi.org/10.1371/journal.pone.0174755

19) Duss, S. B., Seiler, A., Schmidt, M. H., Pace, M., Adamantidis, A., Müri, R. M., & Bassetti, C. L. (2017). The role of sleep in recovery following ischemic stroke: a review of human and animal data. *Neurobiology of sleep and circadian rhythms*, *2*, 94–105. doi: https://doi.org/10.1016/j.nbscr.2016.11.003

20) Sterr, A., Kuhn, M., Nissen, C., Ettine, D., Funk, S., Feige, B., . . . Riemann, D. (2018). Post-stroke insomnia in community-dwelling patients with chronic motor stroke: Physiological evidence and implications for stroke care. *Scientific Reports*, *8*(1), 1–9. doi: https://doi.org/10.1038/s41598-018-26630-y

21) Khot, S. P., & Morgenstern, L. B. (2019). Sleep and stroke. Stroke, 50(6), 1612–1617. doi: https://doi.org/10.1161/STROKEAHA.118.023553

22) Fleming, M. K., Smejka, T., Henderson Slater, D., Chiu, E. G., Demeyere, N., & Johansen- Berg, H. (2021). Self-reported and objective sleep measures in stroke survivors with incomplete motor recovery at the chronic stage. *Neurorehabilitation and Neural Repair*, *35*(10), 851–860. doi: https://doi.org/10.1177/15459683211029889

23) Fleming, M. K., Smejka, T., Henderson Slater, D., van Gils, V., Garratt, E., Yilmaz Kara, E., & Johansen-Berg, H. (2020). Sleep disruption after brain injury is

For peer review only

For peer review only - http://bmjopen.bmj.com/site/about/guidelines.xhtml

associated with worse motor outcomes and slower functional recovery. *Neurorehabilitation and Neural Repair*, *34*(7), 661–671. doi: https://doi.org/10.1177/154596832092966

24) Fulk, G. D., Boyne, P., Hauger, M., Ghosh, R., Romano, S., Thomas, J., . . . Klingman, K. (2020). The impact of sleep disorders on functional recovery and participation following stroke: A systematic review and meta- analysis. Neurorehabilitation and Neural Repair, 34(11), 1050–1061. doi: https://doi.org/10.1177/1545968320962501

25) Iddagoda, M. T., Inderjeeth, C. A., Chan, K., & Raymond, W. D. (2020). Post-stroke sleep disturbances and rehabilitation outcomes: a prospective cohort study. Internal Medicine Journal, 50(2), 208–213. doi: https://doi.org/10.1111/imj.14372

26) Espie, C. A., Kyle, S. D., Hames, P., Gardani, M., Fleming, L., & Cape, J. (2014). The sleep condition indicator: a clinical screening tool to evaluate insomnia disorder. *BMJ open*, *4*(3), e004183. doi: doi:10.1136/bmjopen-2013-004183

27) Espie, C. A., Farias Machado, P., Carl, J. R., Kyle, S. D., Cape, J., Siriwardena, A. N., & Luik, A. I. (2018). The sleep condition indicator: reference values derived from a sample of 200 000 adults. *Journal of sleep research*, *27*(3), e12643. doi: https://doi.org/10.1111/jsr.12643

28) Robertson, E. M., Pascual-Leone, A., & Press, D. Z. (2004). Awareness modifies the skill-learning benefits of sleep. *Current biology*, *14*(3), 208–212. doi: https://doi.org/10.1016/j.cub.2004.01.027

29) Lyle, R. C. (1981). A performance test for assessment of upper limb function in physical rehabilitation treatment and research. International journal of rehabilitation research, 4(4), 483–492.

30) Fugl-Meyer, A. R., Jääskö, L., Leyman, I., Olsson, S., & Steglind, S. (1975). A method for evaluation of physical performance. *Scand. J. Rehabil. Med*, *7*(1), 13–31.

31) Mathiowetz, V., Weber, K., Kashman, N., & Volland, G. (1985). Adult norms for the nine hole peg test of finger dexterity. *The Occupational Therapy Journal of Research*, *5*(1), 24–38. doi: https://doi.org/10.1177/153944928500500102

32) Collen, F. M., Wade, D. T., Robb, G., & Bradshaw, C. (1991). The Rivermead mobility index: a further development of the Rivermead motor assessment. *International disability studies*, *13*(2), 50–54. doi: https://doi.org/10.3109/03790799109166684

33) Kroenke, K., Spitzer, R. L., & Williams, J. B. (2001). The PHQ-9: validity of a brief depression severity measure. Journal of general internal medicine, 16(9), 606–613. doi: https://doi.org/10.1046/j.1525-1497.2001.016009606.x

34) Chung, F., Abdullah, H. R., & Liao, P. (2016). Stop-bang questionnaire: a practical approach to screen for obstructive sleep apnea. *Chest*, *149*(3), 631–638. doi: https://doi.org/10.1378/chest.15-0903

For peer review only - http://bmjopen.bmj.com/site/about/guidelines.xhtml

35) Wilson, S. J., Nutt, D., Alford, C., Argyropoulos, S., Baldwin, D. S., Bateson, A., . . . others (2010). British association for psychopharmacology consensus statement on evidence-based treatment of insomnia, parasomnias and circadian rhythm disorders. Journal of psychopharmacology, 24(11), 1577–1601. doi: https://doi.org/10.1177/0269881110379307

36) Stinear, C. M., Byblow, W. D., Ackerley, S. J., Smith, M.-C., Borges, V. M., & Barber, P. A. (2017). Prep2: A biomarker-based algorithm for predicting upper limb function after stroke. *Annals of clinical and translational neurology*, *4*(11), 811–820. doi: https://doi.org/10.1002/acn3.488

37) Smith, M.-C., Ackerley, S. J., Barber, P. A., Byblow, W. D., & Stinear, C. M. (2019). Prep2 algorithm predictions are correct at 2 years poststroke for most patients. Neurorehabilitation and neural repair, 33(8), 635–642. doi: https://doi.org/10.1177/1545968319860481

38) Fleming, M. K., Smejka, T., Macey, E., Luengo-Fernandez, R., Henry, A. L., Robinson, B., . . . Johansen-Berg, H. (2023). Improving sleep after stroke: a randomised controlled trial of digital cognitive behavioural therapy for insomnia. *medRxiv*, 2023–02. doi: https://doi.org/10.1101/2023.02.07.23285580

39) Weightman, M., Robinson, B., Fallows, R., Henry, A. L., Kyle, S. D., Garratt, E., ... others (2023). Improving sleep and learning in rehabilitation after stroke, part 2 (inspires2): study protocol for a home-based randomised control trial of digital cognitive behavioural therapy (dcbt) for insomnia. *BMJ open*, *13*(4), e071764. doi: http://dx.doi.org/10.1136/bmjopen-2023-071764

40) Fleming, M. K., Newham, D. J., & Rothwell, J. C. (2018). Explicit motor sequence learning with the paretic arm after stroke. *Disability and Rehabilitation*, *40*(3), 323–328. doi: https://doi.org/10.1080/09638288.2016.1258091

41) Arnal, P. J., Thorey, V., Debellemaniere, E., Ballard, M. E., Bou Hernandez, A., Guillot, A., . . . others (2020). The dreem headband compared to polysomnography for electroencephalographic signal acquisition and sleep staging. *Sleep*, *43*(11), zsaa097. doi: https://doi.org/10.1093/sleep/zsaa097

42) Jafarzadeh Esfahani, M., D. Weber, F., Boon, M., Anthes, S., Almazova, T., van Hal, M., ... & Dresler, M. (2023). Validation of the sleep EEG headband ZMax. *bioRxiv*, 2023-08.

43) Rossi, S., Antal, A., Bestmann, S., Bikson, M., Brewer, C., Brockmöller, J., ... others (2021). Safety and recommendations for TMS use in healthy subjects and patient populations, with updates on training, ethical and regulatory issues: Expert guidelines. *Clinical Neurophysiology*, *132*(1), 269–306. doi: https://doi.org/10.1016/j.clinph.2020.10.003

44) Zeiler, S. R., & Krakauer, J. W. (2013). The interaction between training and plasticity in the post-stroke brain. *Current opinion in neurology*, *26*(6), 609. doi: 10.1097/WCO.0000000000000025

For peer review only

For peer review only

For peer review only - http://bmjopen.bmj.com/site/about/guidelines.xhtml

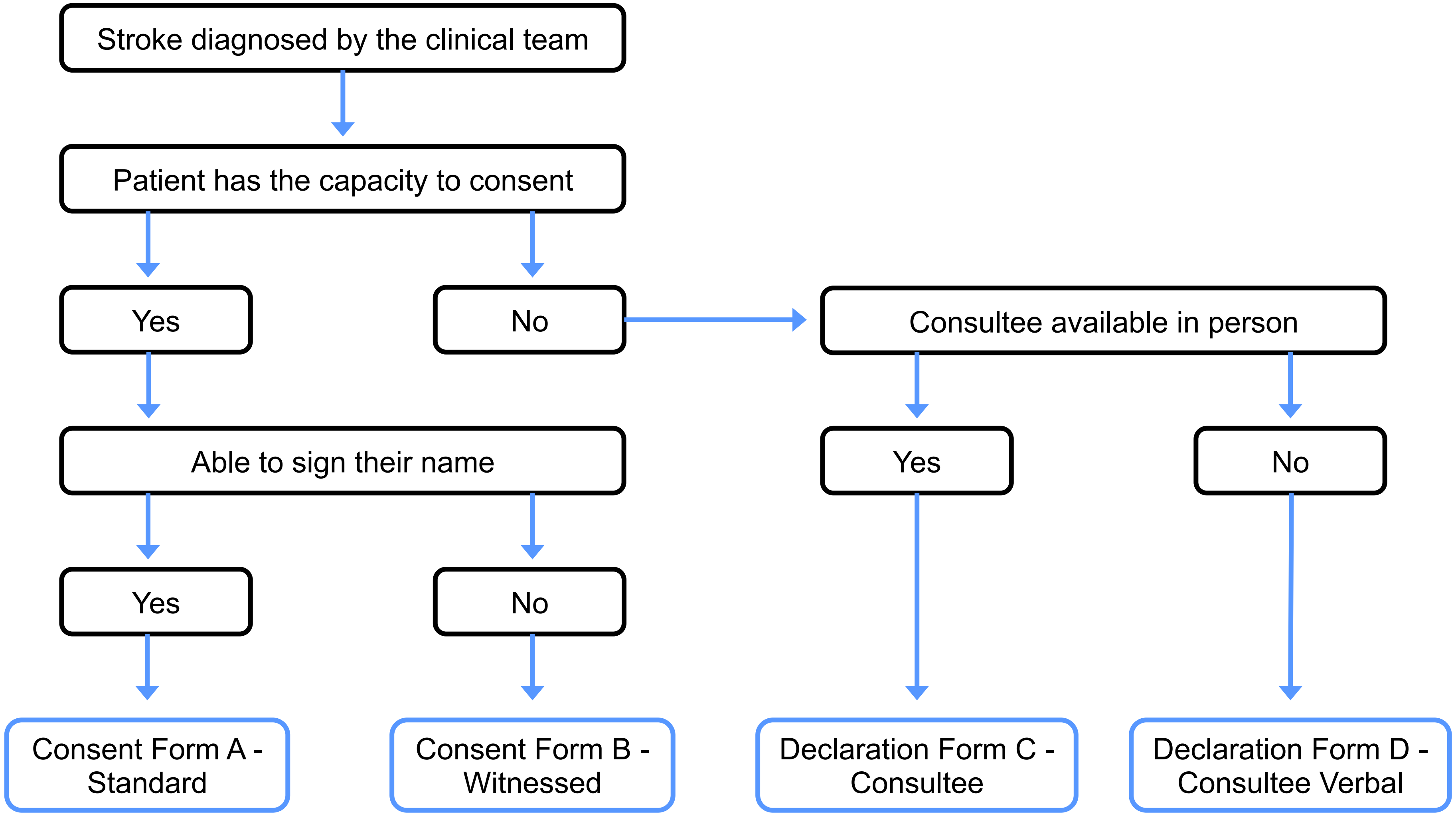
Stroke diagnosed by the clinical team
Patient has the capacity to consent
Yes
No
Consultee available in person
Able to sign their name
Yes
No
Yes
No
Consent Form A - Standard
Consent Form B - Witnessed
Declaration Form C - Consultee
Declaration Form D - Consultee Verbal

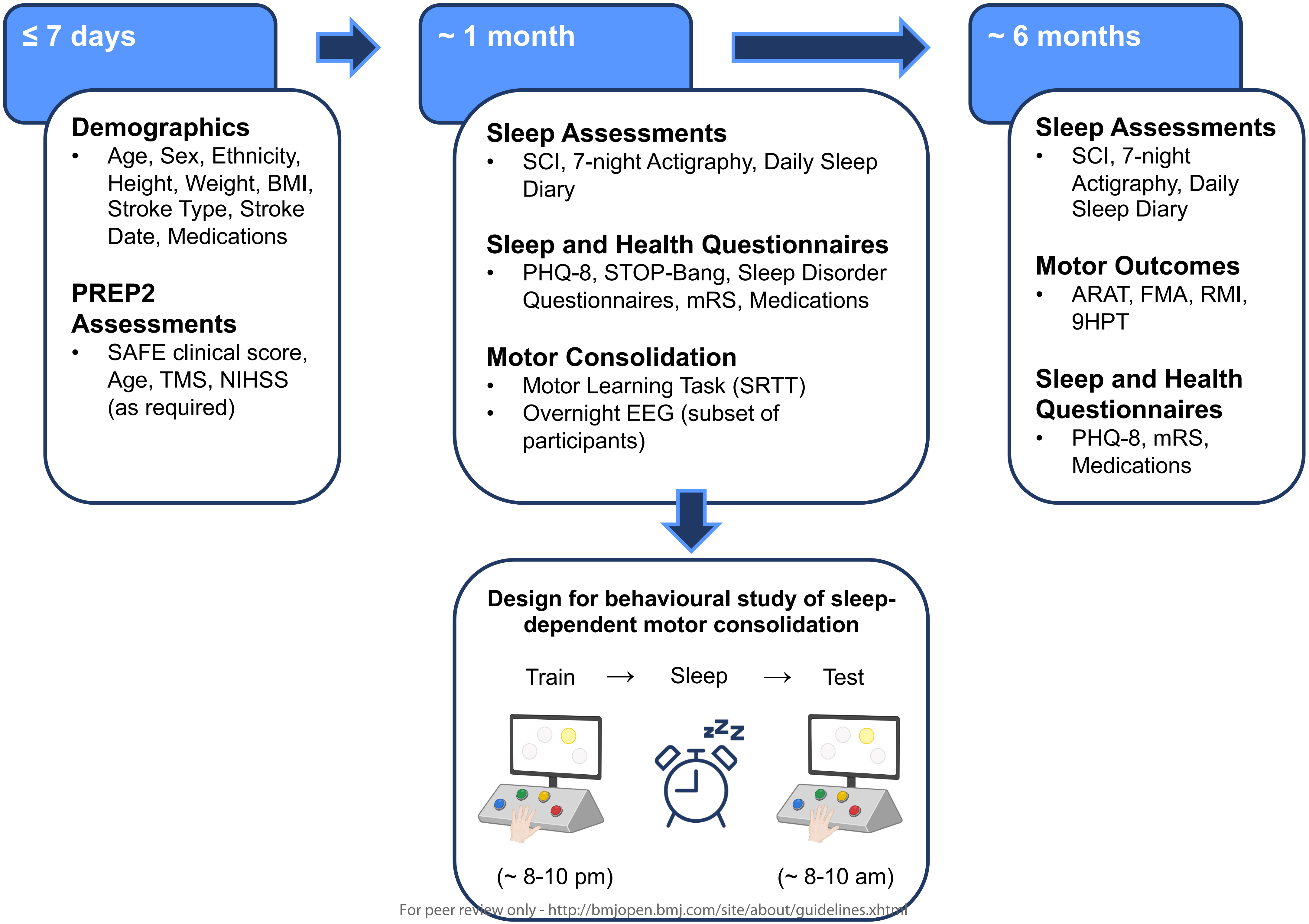
≤ 7 days
Demographics
Age, Sex, Ethnicity, Height, Weight, BMI, Stroke Type, Stroke Date, Medications
PREP2 Assessments
SAFE clinical score, Age, TMS, NIHSS (as required)
~ 1 month
Sleep Assessments
SCI, 7-night Actigraphy, Daily Sleep Diary
Sleep and Health Questionnaires
PHQ-8, STOP-Bang, Sleep Disorder Questionnaires, mRS, Medications
Motor Consolidation
Motor Learning Task (SRTT)
Overnight EEG (subset of participants)
~ 6 months
Sleep Assessments
SCI, 7-night Actigraphy, Daily Sleep Diary
Motor Outcomes
ARAT, FMA, RMI, 9HPT
Sleep and Health Questionnaires
PHQ-8, mRS, Medications
Design for behavioural study of sleep-dependent motor consolidation
Train → Sleep → Test
(~ 8-10 pm)
(~ 8-10 am)

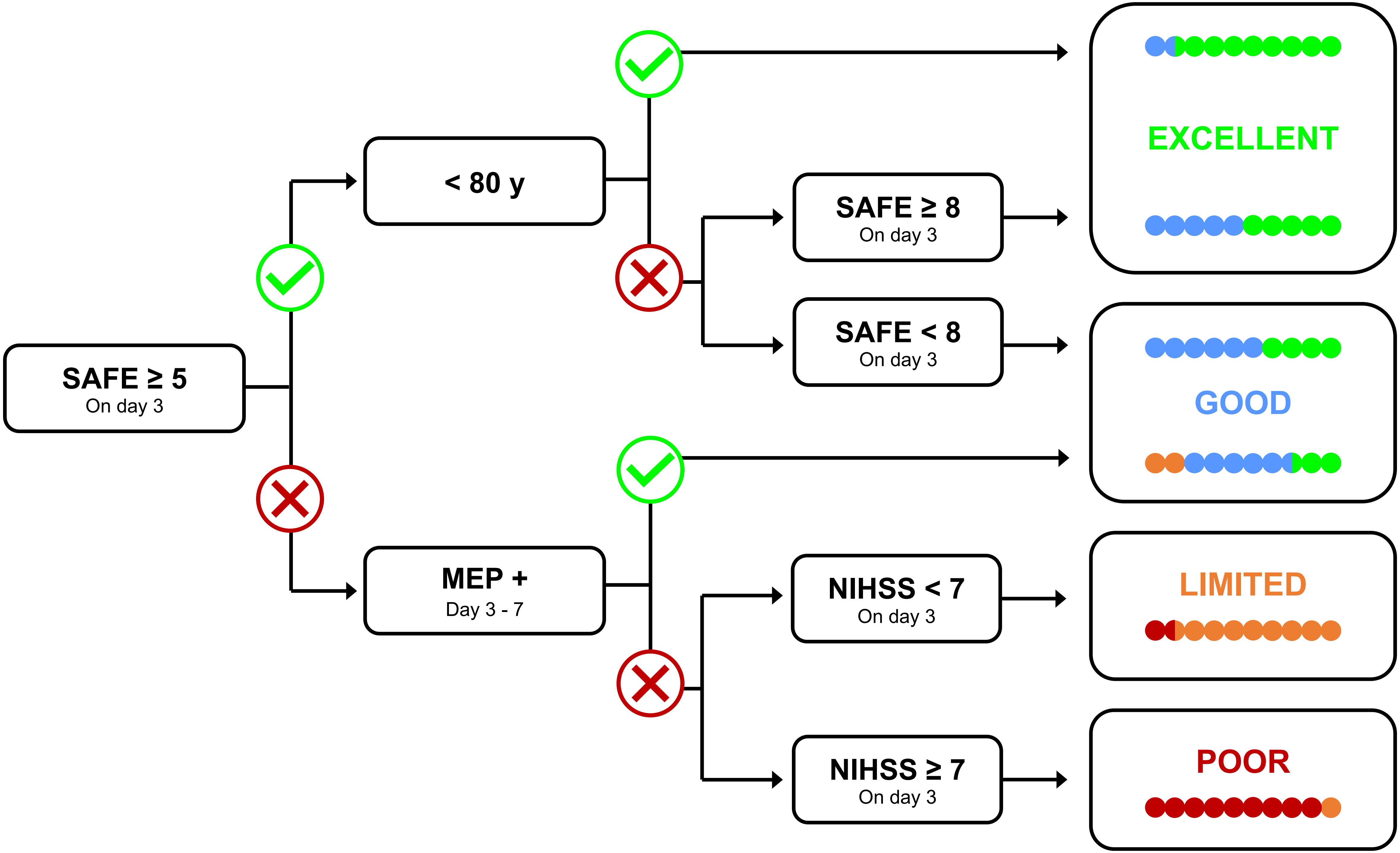
SAFE ≥ 5
On day 3
< 80 y
MEP +
Day 3 - 7
SAFE ≥ 8
On day 3
SAFE < 8
On day 3
NIHSS < 7
On day 3
NIHSS ≥ 7
On day 3
EXCELLENT
GOOD
LIMITED
POOR

Supplementary File 1: Button box technical note

Overview

The button box was designed with a button layout based on the specifications given by Gudberg, Wulf & Johansen-Berg[1] using commercially available parts.

Following initial evaluation, the design was revised to reduce button noise to minimise any potential for interference.

The button box communicates with the experiment software via a programmable USB-Human Interface Device (HID) module. This allows the buttons to be presented as any keyboard or joystick button required by the experiment software without the need for any additional software or drivers and is compatible with all modern operating systems.

Construction

The button box is built around an appropriately sized aluminium instrument case (CamdenBoss RTM2703-BR/B, RS stock no. 170-7825) with a sloped front on which four 60mm buttons (low-profile domed arcade buttons, purchased from Arcade World UK) are mounted according to the layout described in Gudberg et al.[1] such that they can be easily operated by the participant while resting their elbow on a flat surface, if needed (Figure 1).

The buttons are connected to a programmable USB HID interface board (Ultimarc U-HID Nano) which is wrapped with heatshrink to provide insulation and mechanical protection and mounted internally to the box. The module's USB interface is then connected to a panel mount USB B socket (RS stock no. 874-1248) on the rear of the case via a short USB cable secured in place with heatshrink.

Noise reduction

The following steps were taken to minimise acoustic noise from the buttons:

The case was lined with sound deadening material (Teroson Adhesive bitumen acoustic insulation, RS stock no. 369-4715) in order to reduce the transmission of acoustic noise via the case.

The stock microswitches provided with the buttons were replaced with a quieter type (Cherry D44X, purchased from Arcade World UK) to reduce noise caused by switch actuation.

Thin self-adhesive foam was added to the back of the button plungers to reduce noise caused by impact of the plunger against the button housing.

For peer review only

A layer of the same sound deadening material used to line the case was applied to the back of the light diffuser and the opposing surface inside the button plunger to deaden sound conducted through the plunger.

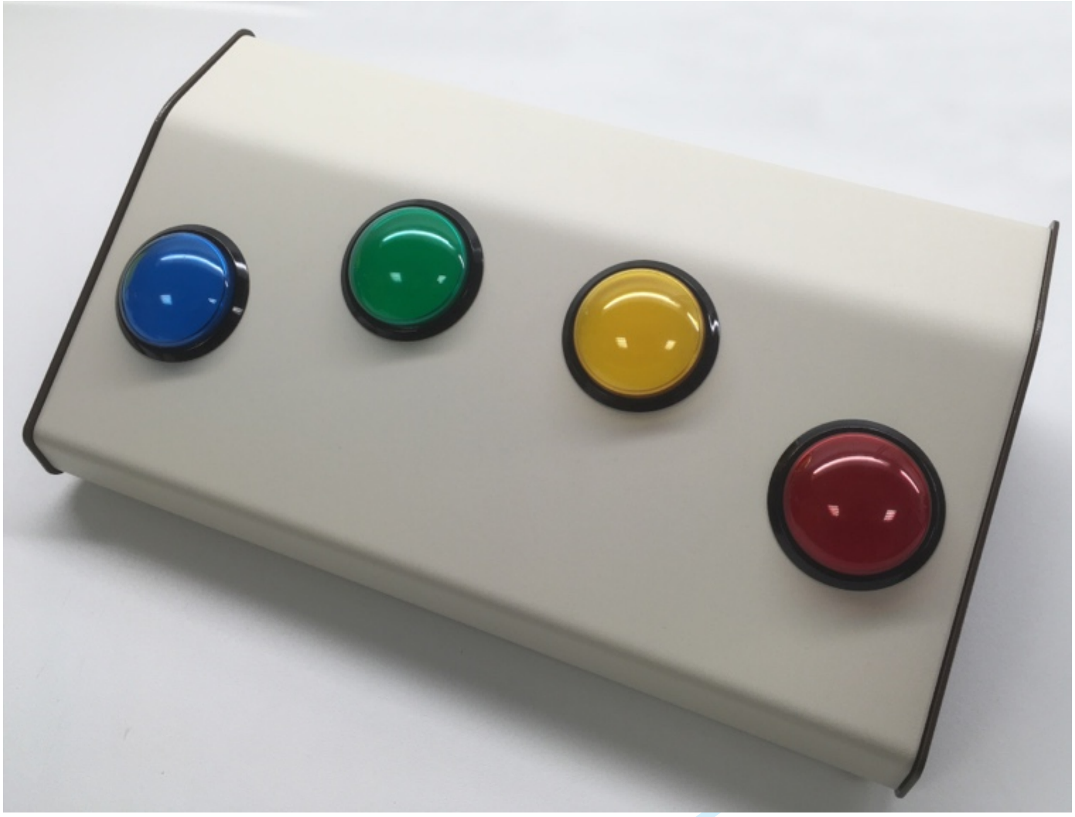

Figure 1: Image of the button box

References

Gudberg, C., Wulff, K., & Johansen-Berg, H. (2015). Sleep-dependent motor memory consolidation in older adults depends on task demands. Neurobiology of aging, 36(3), 1409–1416. doi: https://doi.org/10.1016/j.neurobiolaging.2014.12.014

For peer review only - http://bmjopen.bmj.com/site/about/guidelines.xhtml
